# Energy metabolism as therapeutic target for aged wound repair by engineered extracellular vesicle

**DOI:** 10.1126/sciadv.adl0372

**Published:** 2024-04-12

**Authors:** Yu Zhuang, Shengjie Jiang, Xiaoling Deng, An Lao, Xiaolin Hua, Yun Xie, Lingyong Jiang, Xudong Wang, Kaili Lin

**Affiliations:** ^1^ Department of Oral and Cranio-maxillofacial Surgery, Shanghai Ninth People’s Hospital, Shanghai Jiao Tong University School of Medicine; College of Stomatology, Shanghai Jiao Tong University; National Center for Stomatology; National Clinical Research Center for Oral Diseases; Shanghai Key Laboratory of Stomatology; Shanghai Research Institute of Stomatology; Research Unit of Oral and Maxillofacial Regenerative Medicine, Chinese Academy of Medical Sciences, Shanghai, China.; ^2^ Obstetrics Department, Shanghai First Maternity and Infant Hospital, School of Medicine, Tongji University, Shanghai, China.; ^3^ Department of Plastic and Reconstructive Surgery, Shanghai Ninth People’s Hospital affiliated to Shanghai Jiao Tong University School of Medicine, Shanghai, China.

## Abstract

Aging skin, vulnerable to age-related defects, is poor in wound repair. Metabolic regulation in accumulated senescent cells (SnCs) with aging is essential for tissue homeostasis, and adequate ATP is important in cell activation for aged tissue repair. Strategies for ATP metabolism intervention hold prospects for therapeutic advances. Here, we found energy metabolic changes in aging skin from patients and mice. Our data show that metformin engineered EV (Met-EV) can enhance aged mouse skin repair, as well as ameliorate cellular senescence and restore cell dysfunctions. Notably, ATP metabolism was remodeled as reduced glycolysis and enhanced OXPHOS after Met-EV treatment. We show Met-EV rescue senescence-induced mitochondria dysfunctions and mitophagy suppressions, indicating the role of Met-EV in remodeling mitochondrial functions via mitophagy for adequate ATP production in aged tissue repair. Our results reveal the mechanism for SnCs rejuvenation by EV and suggest the disturbed energy metabolism, essential in age-related defects, to be a potential therapeutic target for facilitating aged tissue repair.

## Teaser

The disturbed ATP metabolism could be a potential target for rescuing senescent cells and improving aged tissue repair.

## INTRODUCTION

Skin, acting as the critical physical barrier to the external stimulus, can protect the body from dehydration, ultraviolet radiation, and pathogen infections (*1*). Considering the indispensable role in maintaining appropriate tissue homeostasis, the repair capacity of skin is key (*2*). However, wound repair competence progressively fails with age in multiple types of tissues and organs (*3*, *4*). Aging skin, vulnerable to age-related functional defects, is unable to trigger a timely wound healing response when exposed to injury (*5*). Poor wound repair of aged adults leads to decreased health span and life span, which have been a challenge for over a century (*2*). Thus, understanding the molecular underpinnings in aging skin is critical to advance therapeutic strategies for ideal wound healing, antagonizing the age-related declines.

Senescent cells (SnCs) accumulate in tissues with aging, contributing to the development of age-related pathologies and declines, mainly via senescence-associated secretory phenotype (SASP) (*6*–*8*). Through secreting proinflammatory and tissue-remodeling factors, SnCs communicate with surroundings and fuel a chronic inflammatory microenvironment in the body, which seriously impedes the damaged tissue repair (*9*, *10*). Moreover, SnCs are characterized with DNA damage, cell cycle arrest, metabolic changes, mitochondria dysfunctions, and reduced autophagy, underlying the impaired capacity for wound repair in aging tissues (*8*, *11*, *12*).

Metabolic regulation in SnCs is essential for tissue homeostasis and for competence to activate repair process in damaged tissue (*13*, *14*). It has been reported that serial passage or radiation-induced senescent fibroblasts accumulate during culture and gradually adopt a more glycolytic phenotype, which indicate SnCs associated with the increased glycolysis state (*15*, *16*). A shift to a more glycolytic phenotype in metabolic profiling tends to contribute to a less energetic state and replication-induced cell senescence (*17*). Furthermore, adenosine diphosphate/adenosine monophosphate to adenosine triphosphate (ATP) ratio is increased in SnCs, which may induce a senescence arrest (*18*). Adequate ATP production is essential in cell activation and functioning for tissue repair, but the metabolic shift in SnCs can result in an energy equivalent deficiency for intracellular anabolism (*19*, *20*). Energy metabolic process is involved in multiple networks and delivery of single specific factor could perturb the inherent pathways, which may inversely exert adverse effects on cell behavior (*21*). In addition, direct administration of exogenous ATP plays little role in cellular metabolism (*22*). Thus, strategies for ATP metabolism intervention hold prospects for therapeutic advances in aging tissue repair.

Mitochondria dysfunction serves as the metabolic driver of senescence, as well as the major regulator of age-related decline (*12*, *23*). Tricarboxylic acid (TCA) cycle and oxidative phosphorylation (OXPHOS), the main efficient energy metabolic process for ATP production via the mitochondrial electron transport chain, take place in mitochondria (*24*). However, mitochondria dysfunction destroys the redox homeostasis, resulting in accumulated cytosolic NADH, and the reduced NAD^+^/NADH ratio leads to ATP depletion and cell cycle arrest (*25*, *26*). When exposed to injury, autophagy is induced to meet cell proliferative and metabolic requirements (*27*), and ablation of autophagy disturbs mitochondrial function as well as ATP generation (*28*, *29*). Autophagy activity descends with aging, resulting in the accumulation of damaged organelles, including mitochondria (*14*), which may account for the defective capacity in wound repair (*30*). It was reported that mitophagy-induced mitochondrial elimination could ameliorate cell senescence and inhibit secretion of SASP factors (*31*).

Extracellular vesicle (EV) therapies have shown emerging prospects in aging fields over recent decades. EV, mediating the communication of bioactive molecules (proteins, small RNAs, and DNA) between cells, can be secreted by almost all cell types (*32*). Mesenchymal stem cells (MSCs) have regenerative capacity, and EV derived from MSCs inherit the intrinsic regenerative potential (*33*). Compared to MSCs, EV have better stability, lower immunogenicity, and no risk of aneuploidy, which make it widely applied in tissue regeneration after various types of damage (*34*). Specifically, EV therapies have proven potential for ameliorating age-related diseases. EV therapies can ameliorate age-related cellular senescence and decrease reactive oxygen species (ROS) accumulation (*35*, *36*), rejuvenate SnCs, and rescue age-related declines (*37*–*39*), as well as potentially modulating mitochondria functions and inflammation microenvironments (*40*, *41*). While the effect of EV therapies in regulating metabolic disorders for specific treatment is limited, engineered EV can be a potential candidate. Metformin is applied in enhancing autophagy and normalizing mitochondrial function, with prospects to be a tool for aging target (*42*, *43*), but direct delivery of metformin has a short half-life period and low bioavailability. Considering the ideal therapeutic potential of metformin in aging-related diseases, and the requirement for strengthening therapeutic efficiency of EV, engineering EV with metformin is an efficient strategy for coupling advantages of both.

Here, we identified the metabolic changes in aging, explored whether metformin engineered EV (Met-EV) can improve aged wound repair via reprogramming metabolism, and uncovered a therapeutical pathway that connects EV, autophagy, mitochondria function, and energy metabolism to skin aging and wound repair.

## RESULTS

### Metabolism is remodeled in aging skin

**Fig. 1.**
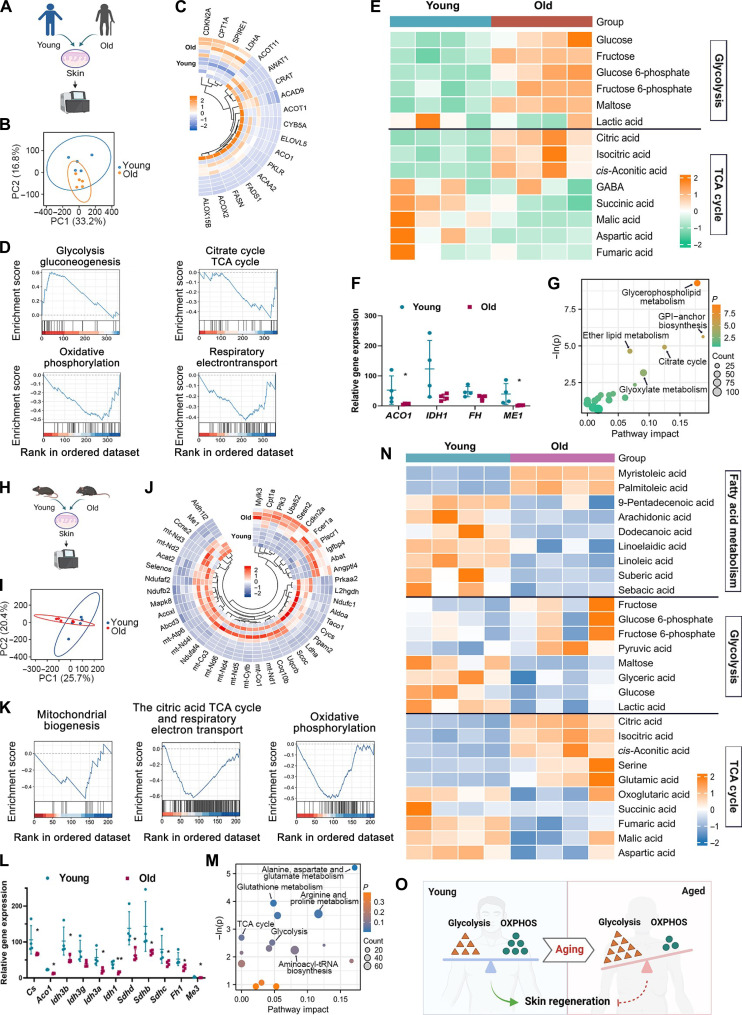
Energy metabolism is reprogrammed in aging skin. (**A** and **H**) Schematic illustration of tissue collection from human or mice. (**B** and **I**) PCA of genes in young and old skin tissues. (**C** and **J**) Circular heatmap displaying differentially expressed genes related to glycolysis, TCA cycle, OXPHOS, fatty acid metabolism, cellular senescence, inflammation, and autophagy. (**D** and **K**) GSEA was conducted to contrast the gene sets associated with glycolysis, TCA cycle, and OXPHOS between young and old skin tissues. (**F** and **L**) Differentially expressed enzymes involved in TCA cycle. (**G** and **M**) Pathway analysis for the effect of aging on skin tissues related to differentially abundant metabolite-related reactions using MetaboAnalyst. (**E** and **N**) Heatmap represented differentially detected metabolites in young and old groups associated with glycolysis, TCA cycle, and fatty acid metabolism. (A) to (G) represented the analysis based on tissues collected from human, and (H) to (N) represented that from mice. (**O**) Schematic of energy metabolism disorder in aging. *n* = 5 was analyzed and shown here. GABA, γ-aminobutyric acid.

To investigate the gene expression and metabolism changes in aging skin, skin tissues from young and old clinical donors (age over 65 years old as old, under 20 years old as young) and mice (24 to 25 months as old, 2 to 3 months as young) were collected and sequenced. From transcriptomic analysis, gene expressions related to cellular energy metabolism were changed in aging skin from both clinical and mouse samples, compared with young skin (Fig. 1, B to D and J to K). Principal components analysis (PCA) displayed the gene distinctly distributed between young and old groups from human and mice in a multivariate way (Fig. 1, B and I). A panel of differentially expressed genes (DEGs) associated with glycolysis and OXPHOS (like *Ldha*, *Cpt1a*, *Taco1*, *mt-Nd*, *mt-Co1*, *Ndufc*, and *Ndufaf* in mice and *LDHA*, *CPT1A*, *PKLR*, *ACAD9*, *ACO1*, *ACAA2*, and *CYB5A* in human), fatty acid metabolism (like in *Sesn2* and *Acoxl* in mice and *ACOT11*, *AWAT1*, and *ACOX2* in human), cellular senescence (like *Cdkn2a*, *Abat*, and *Ccne2* in mice and *CDKN2A* in human), inflammation (*Mylk3*, *Fcer1a*, and *Plscr1* in mice), and autophagy (*Scoc*, *Uba52*, and *Plk3* in mice) were displayed (Fig. 1, C and J). Kyoto Encyclopedia of Genes and Genomes (KEGG) and Gene Ontology (GO) analysis showed that pathways enriched from DEGs were clustered in metabolic processes (fig. S1, B, C, G, and H). The gene set enrichment analysis (GSEA) showed that compared with young skin, old skin exhibited increased glycolysis activity but inhibited TCA and OXPHOS activity and mitochondria biogenesis, which was consistent in both mice and human skin tissues (Fig. 1, D and K). To further verify the expressions of key molecules, reverse transcription quantitative polymerase chain reaction (RT-qPCR) and Western blot were conducted to analyze the metabolic changes in aged skin tissues. The results showed the changes of TCA cycle–related key enzymes in old tissues, including the protein expression inhibition of FH1, IDH3g, and ME1 in old skin tissues from both clinical and animal samples (fig. S1, D and I). Moreover, the mRNA expression of *FH*, *IDH1*, *ME1*, and *ME2* was lower in old human skin tissues (fig. S1E), and expression of *FH*, *IDH1*, *IDH3g*, and *ME1* was reduced in old mouse tissues (fig. S1J).

For metabolomic analysis, the component analysis of metabolites showed the changes in energy metabolism (Fig. 1, E, G, N, and M). Not all the metabolites related to glycolysis were increased or related to TCA cycle were reduced in aging skin (Fig. 1, E and N), which can be elaborated by the dysregulated enzyme activities. We found that expressions of certain enzymes (*ACO1* and *ME1* in human and *Cs*, *Aco1*, *Idh3b*, *Idh3a*, *Idh1*, *Sdhd*, *Sdhb*, *Sdhc*, *Fh1*, and *Me3* in mice) were significantly inhibited in aging skin, and these key enzymes play a critical role in the regulating process of TCA cycle, for instance, inhibited expression of *Idh*, *Sdh*, and *Fh* induced reduced oxoglutaric acid, fumaric acid, and malic acid, respectively (Fig. 1, F and L), which indicated that the dysregulated key enzymes could perturb TCA cycle. Then, integrative pathway analysis was performed and results showed that aging exerted influence on metabolism, and a metabolic shift (increased glycolysis and inhibited citrate cycle), indicated as Warburg effect, was observed (Fig. 1, G and M, and fig. S1, A and F).

In summary, the transcriptomic and metabolomic results indicated that metabolism was reprogrammed in aging skin compared to young skin, especially balance in energy metabolism (glycolysis, TCA cycle, and OXPHOS) was broken (Fig. 1O).

### MSCs are activated to secrete bioactive EV via metformin treatment

**Fig. 2.**
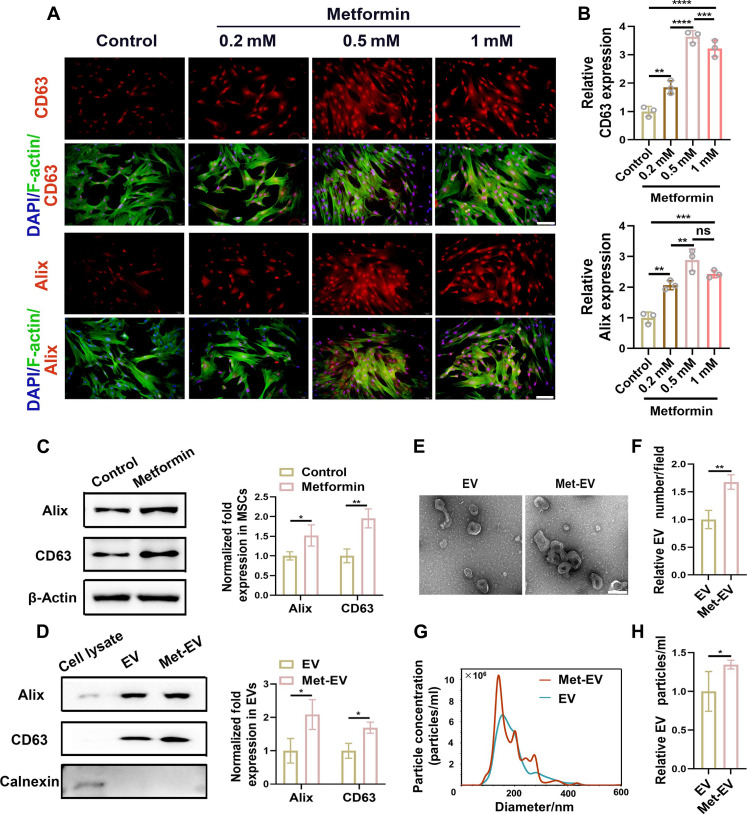
Generation and characterization of Met-EV. (**A** and **B**) Influence of different concentrations of metformin on MSCs for 48 hours via fluorescence staining (scale bars: 100 μm) and semi-quantitative analysis. Representative immunoblot (left) and quantification (right) for expression of Alix and CD63 in MSCs [(**C**), β-actin serving as internal control] and MSC-derived EV [(**D**), calnexin serving as negative control]. (**E** and **F**) TEM image of Met-EV (scale bar: 200 nm) and quantitative analysis of EV numbers. (**G** and **H**) NTA analysis of EV and Met-EV and particle concentrations. *n* = 3 was analyzed and shown here. EV derived from MSCs were as indicated as EV, and EV from metformin-treated MSCs were indicated as Met-EV. DAPI, 4′,6-diamidino-2-phenylindole; ns, not significant.

To determine the effect of metformin on MSCs, cell counting kit-8 assay was used to examine the proliferation of cells incubated with metformin, and results showed that when concentration was lower than 0.5 mM, metformin promoted cell proliferation with concentration augmented and reached the peak proliferative capacity at 0.5 mM (fig. S2A). Fluorescence (Fig. 2, A and B) and Western blotting (Fig. 2C) results showed that protein expression of Alix and CD63 in MSCs was increased the most significantly when concentration was at 0.5 mM. Furthermore, immunoblot for EV proteins suggested that Met-EV expressed more characteristic proteins (Alix and CD63) than EV, indicating the successful isolation of EV (Fig. 2D). Typical goblet vesicle structure of EV and Met-EV can be observed from transmission electron microscopy (TEM) results (Fig. 2, E and F), and the size and concentration distribution were analyzed using nanoparticle tracking analysis (NTA) (Fig. 2, G and H). The increased number and concentration of Met-EV compared with EV indicated that metformin could improve EV yield from MSCs.

To determine whether EV carried metformin after MSCs were treated with the drug, the ultraviolet absorption curves of metformin, EV, EV after sonication, Met-EV, and Met-EV solutions were analyzed, and the results suggested that metformin showed a small characteristic peak in the range of 200- to 300-nm wavelength but with no characteristic peak in EV groups, indicating that metformin is not the cargo in Met-EV (fig. S2B).

To further make clear the changes that metformin treatment made on EV cargos, we conducted the quantitative proteome analysis for proteins in EV and Met-EV, and the result showed the differentially detected proteins in EV and Met-EV groups associated with mitochondrial related metabolism, proteins of which are involved in mitochondrial related pathways (like RPS3, IQGAP1, HSP90AA1, COL11A1, NRP1, etc.) or located in subcellular mitochondria (like RPS28, FH, ATP5F1A, CKMT2, ETFA, etc.) (fig. S2C). Moreover, GO and KEGG functional comments (fig. S2D) suggested 62 differentially detected proteins involved in the metabolic pathways, and KEGG enrichment (fig. S2E) showed the changes in metabolism including glycolysis and TCA cycle, result of which indicate the changes related to metabolism in Met-EV cargos.

### Met-EV mitigate cellular senescence and restore dysfunctional SnCs

To figure out whether Met-EV could mitigate cell senescence and restore cell functions, SnCs were constructed and cocultured with EV and Met-EV. Fibroblasts and ECs were exposed to 10-Gy x-rays to induce cellular senescence, and induced senescence phenotype was determined 10 days later, the results of which suggested that senescent fibroblasts (fig. S3, A to C) and ECs (fig. S4, A to C) exhibited increased cell SA-β-Gal activity, expression of senescence (*p21* and *p53*)–and inflammation (*iNOS*, *IL-1*β, and *IL-6*)–related markers, and SASP secretion [interleukin-1β (IL-1β) and IL-6], compared with control cells.

**Fig. 3.**
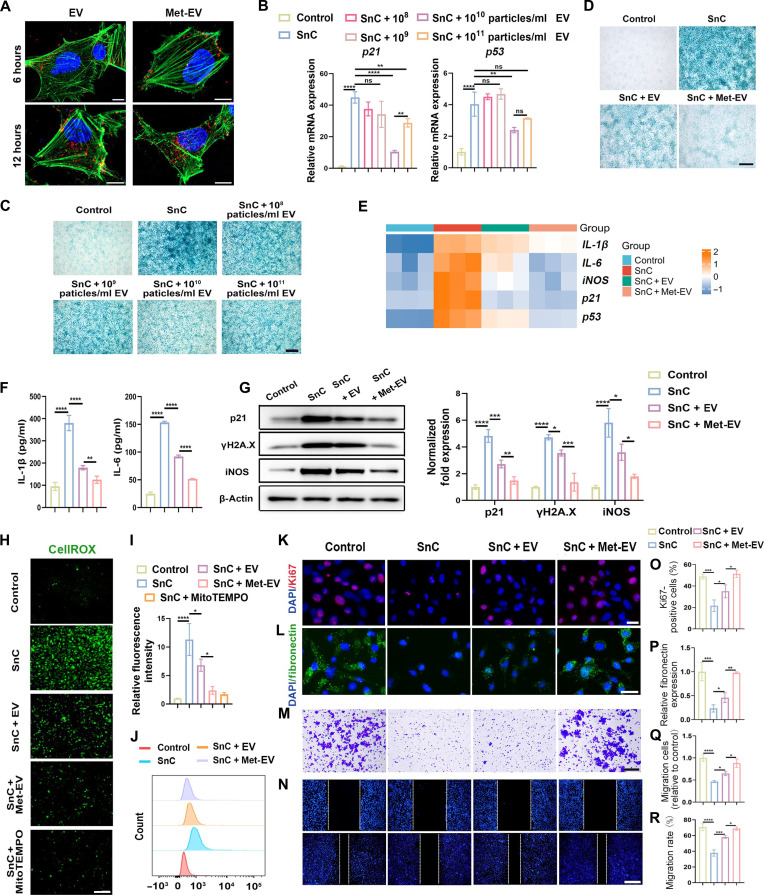
Met-EV mitigate cellular senescence, alleviate inflammation, and promote functions of fibroblasts. (**A**) Images of PKH26-labeled EV or Met-EV uptake by fibroblasts, red indicating PKH26 and green indicating F-actin (scale bars: 10 μm). (**B**) RT-qPCR analysis of senescence (*p21* and *p53*) markers in fibroblasts incubated with EV for 48 hours. Representative images of SA-β-Gal staining (scale bar: 500 μm) of fibroblasts treated with different concentrations of EV for 48 hours (**C**) or different types of EV for 48 hours (**D**). (**E**) Heatmap for RT-qPCR analysis of senescence (*p21* and *p53*)–and inflammation (*iNOS*, *IL-1*β, and *IL-6*)–related markers in fibroblasts. (**F**) Enzyme-linked immunosorbent assay (ELISA) analysis for IL-1β and IL-6 secreted from fibroblasts. (**G**) Representative immunoblot (left) for senescence (p21 and γH2A.X)–and inflammation (iNOS)–related markers in fibroblasts cocultured with EV for 72 hours and quantitative analysis (right). Cellular ROS detection using CellROX staining (scale bar: 400 μm) and quantification (**H** and **I**) and flow cytometry analysis (**J**). Representative images and quantification of Ki67 staining (**K** and **O**) and fibronectin staining (**L** and **P**) in fibroblasts (scale bar: 50 μm). Representative images and quantitative analysis of transwell migration assays (scale bar: 200 μm) (**M** and **Q**) and scratch assays (scale bar: 500 μm) (**N** and **R**) in fibroblasts. *n* = 3 was analyzed and shown here. Control indicated fibroblasts without stimulus, SnC indicated senescence-induced fibroblasts, and EV and Met-EV groups indicated senescence-induced fibroblasts cocultured with EV or Met-EV, respectively.

**Fig. 4.**
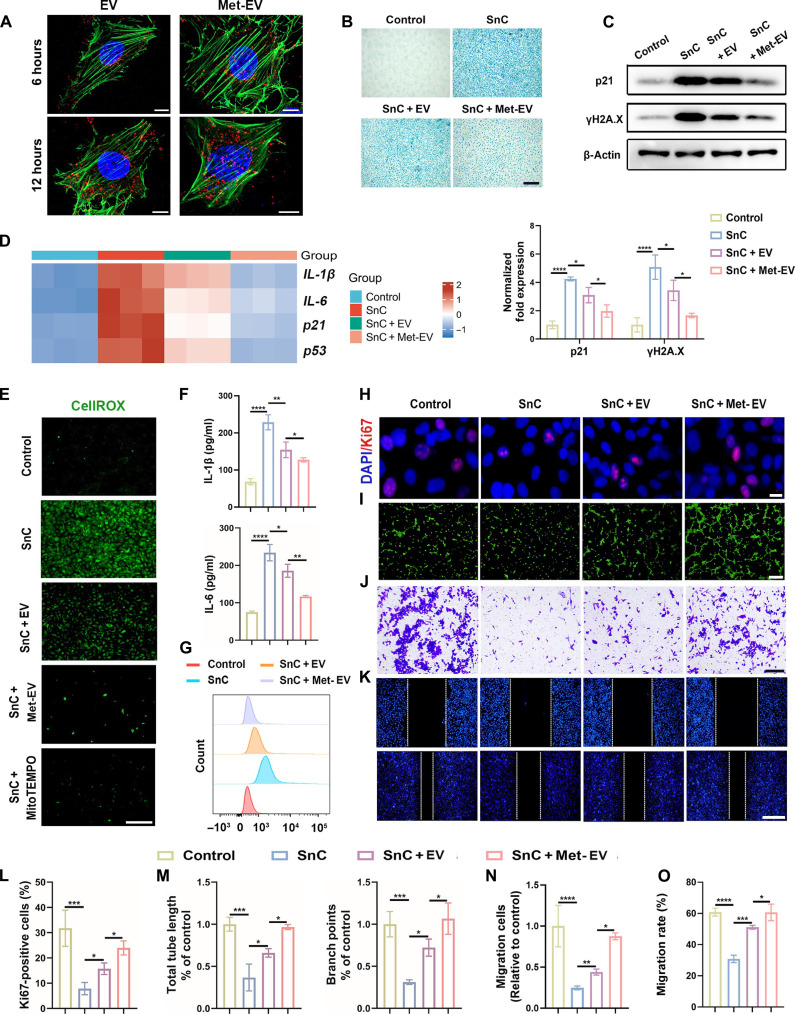
Met-EV mitigate cellular senescence, alleviate inflammation, and promote functions of endothelial cells. (**A**) Images of PKH26-labeled EV or Met-EV uptake by endothelial cells (ECs), red indicating PKH26 and green indicating F-actin (scale bar: 10 μm). (**B**) Representative images of SA-β-Gal staining of ECs incubated with EV for 48 hours (scale bar: 500 μm). (**C**) Representative immunoblot (top) for senescence-related markers (p21 and γH2A.X) in ECs cocultured with EV for 72 hours and quantitative analysis (bottom). (**D**) Heatmap for RT-qPCR analysis of senescence (*p21* and *p53*)–and inflammation (*IL-1*β and *IL-6*)–related markers in ECs cocultured with EV for 48 hours. (**F**) ELISA analysis for IL-1β and IL-6 secreted from ECs. Cellular ROS detection in ECs using CellROX staining (scale bar: 400 μm) (**E**) and flow cytometry analysis (**G**). (**H** and **L**) Representative images and quantification of Ki67 staining in ECs (scale bar: 20 μm). Representative images and quantitative analysis of tube formation assay on Matrigel (scale bar: 300 μm) (**I** and **M**), transwell migration assays (scale bar: 200 μm) (**J** and **N**) and scratch assays (scale bar: 500 μm) (**K** and **O**) in ECs. *n* = 3 was analyzed and shown here. Control indicated ECs without stimulus, SnC indicated senescence-induced ECs, and EV and Met-EV groups indicated senescence-induced ECs cocultured with EV or Met-EV, respectively.

To evaluate the uptake of EV by fibroblasts and endothelial cells, PKH26-labeled EV was coincubated with cells for 6 and 12 h, and results showed that more EV was endocytosed by cells after 12 hours of coculture than 6 hours, and there was no significant difference in fluorescence intensity between EV and Met-EV groups in both fibroblasts (Fig. 3A) or endothelial cells (Fig. 4A). SnCs treated with different concentrations of EV exhibited inhibited SA-β-Gal activity (Fig. 3C), decreased mRNA level expression of senescence markers (*p21* and *p53*) (Fig. 3B). Concentration of EV at 10^10^ particles/ml exhibited the best therapeutic effect in ameliorating cell senescence, and the concentration of which was used in further in vitro experiments (Fig. 3, B and C). Met-EV decreased the SA-β-Gal activity compared with phosphate-buffered saline (PBS) and EV groups, indicating the mitigated senescence phenotype of cells (Figs. 3D and 4B). RT-qPCR and Western blotting results showed that Met-EV rescued the senescence-induced increase of senescence markers p21, p53, and γH2A.X and inflammation markers inducible nitric oxide synthase (iNOS), IL-1β, and IL-6 in fibroblasts (Fig. 3, E and G, and fig. S3D) and ECs (Fig. 4, C and D, and fig. S4D) at mRNA and protein levels. Moreover, Met-EV suppressed SASP secretion like IL-1β and IL-6 both in senescent fibroblasts (Fig. 3F) and ECs (Fig. 4F). In addition, CellROX staining showed that Met-EV could significantly clear senescence-induced ROS, indicating the improved oxidative metabolism (Figs. 3, H to J, and 4, E and G).

To examine cell proliferation, Ki67 staining was performed, the results showing that Met-EV promoted cell proliferation compared to PBS and EV (Figs. 3, K and O, and 4, H and L). Then, cell functions were evaluated. For senescent fibroblasts, protein expression of fibronectin was increased in Met-EV treatment (Fig. 3, L and P). Senescent ECs incubated with Met-EV improved tube formation on Matrigel in vitro (Fig. 4, I and M). Furthermore, Met-EV notably enhanced cell migration ability, as shown in transwell migration assays (Figs. 3, M and Q, and 4, J and N) and scratch assays (Figs. 3, N and R, and 4, K and O). Together, the results indicated that Met-EV significantly ameliorated cellular senescence, as well as rejuvenated dysfunctional fibroblasts and ECs.

### Met-EV promote wound healing in aging mice

**Fig. 5.**
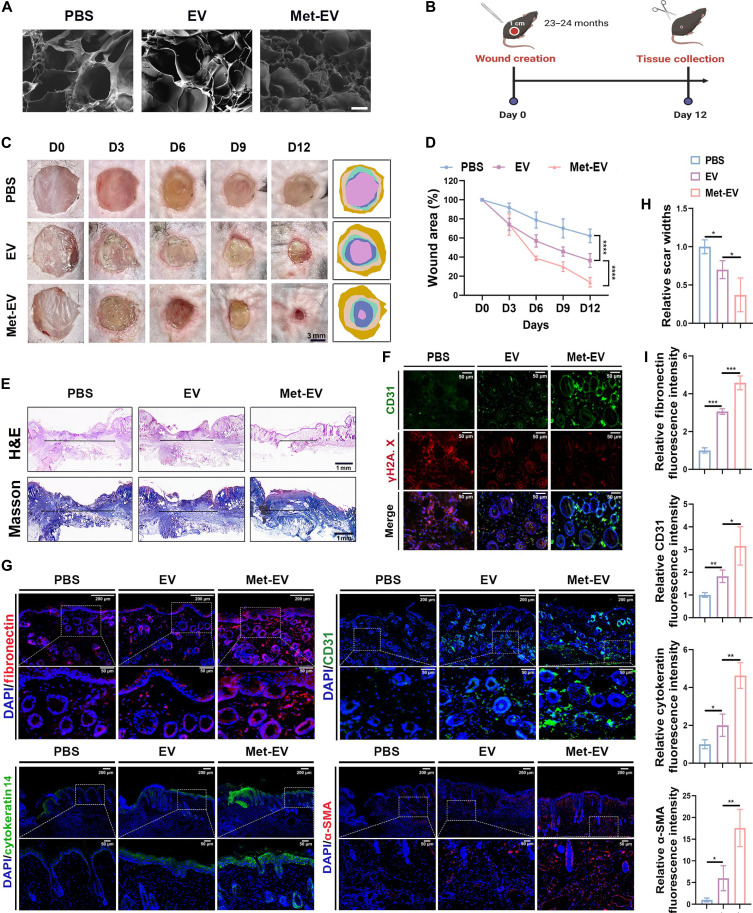
Met-EV promote wound healing and blood vessel formation in aging mice. (**A**) Scanning electron microscopy images for SA hydrogel, with three groups indicating SA hydrogel + PBS, SA hydrogel + EV, and SA hydrogel + Met-EV, respectively (scale bar: 50 μm). (**B**) Schematic illustration of the timeline for skin repair experiments. (**C**) Representative wound closure images in aging skin at each time point after operation treated with EV functionalized hydrogel (scale bar: 3 mm) and corresponding wound area tracing (*n* = 8). (**D**) Quantitative analysis of remaining wound area (*n* = 8). (**E**) H&E and Masson’s trichrome staining of repaired skin tissues at day 12, with horizontal arrows indicating scar width (scale bar: 1 mm), and quantitative analysis of scar widths was shown in (**H**) (*n* = 3). (**F**) Fluorescence costaining of CD31 and γH2A.X in newly born skin tissues (scale bars: 50 μm) (*n* = 3). (**G**) Representative immunofluorescence images of fibronectin (red), cytokeratin 14 (green), CD31 (green), and α-SMA (red) in newly born skin tissues in aging skin treated with hydrogel scaffolds loaded with EV and Met-EV (scale bars: 200 and 50 μm for magnification), and quantification of fluorescence intensity was displayed in (**I**) (*n* = 3).

To explore the effect of EV and Met-EV on wound repair in aging skin, 23- to 24-month-old mice were used. Since nanosized EV were easy to be flushed if directly injected into tissues, the EV/Met-EV functionalized sodium alginate (SA) hydrogel, with porous structure (Fig. 5A) were used as the scaffold for wound repair in aging skin. The results showed that Met-EV significantly promoted wound closure with smaller remaining wound (13.54% relative to origin wound area) at day 12, compared to PBS and EV group (respectively 62.25 and 36.41% remaining area) (Fig. 5, C and D). Hematoxylin and eosin (H&E) and Masson’s trichrome staining results suggested that the minimum wound width was observed in Met-EV group, indicating a higher re-epithelialization rate and lower scar formation rate of Met-EV group (Fig. 5, E and H). Subsequently, Met-EV exhibited the lowest senescence-related γH2A.X and highest angiogenesis-related CD31 expression, compared with PBS and EV group, in costaining assays (Fig. 5F). To evaluate the activity of cell function and formation of blood vessels, immunofluorescence staining was performed, and results showed that Met-EV promoted expression of fibronectin (dermis formation related), cytokeratin 14 (follicle formation related), CD31, and α-SMA (new-born vessel formation related) (Fig. 5, G and I). The above in vivo results revealed that Met-EV could significantly promote the wound repair in aging mice.

To determine whether Met-EV is efficacious in wound repair in general or only helpful for aged mice, the wound healing assays in young mice were conducted. The results showed that both EV and Met-EV significantly promoted wound closure and re-epithelialization rate at day 12, compared to PBS group (fig. S5, B to E); in addition, EV and Met-EV enhanced the expression of follicle generation–related cytokeratin 14 and blood vessel formation–related CD31 and α-SMA (fig. S5F), but there was no significant difference between EV group and Met-EV group.

### Energy metabolism is reprogrammed in Met-EV–mediated aged wound repair via alleviated mitochondria dysfunction

Above in vivo and in vitro experiments have confirmed the therapeutic effect of EV and Met-EV on aged wound repair and cellular senescence, but the relationship between EV and imbalanced energy metabolism in aged skin wound is unknown. To explore the roles of metabolic process in EV-mediated wound healing in aging skin, we performed the transcriptomic and metabolomic analysis of fibroblasts from aged mouse skin tissues treated with PBS, EV, and Met-EV.

**Fig. 6.**
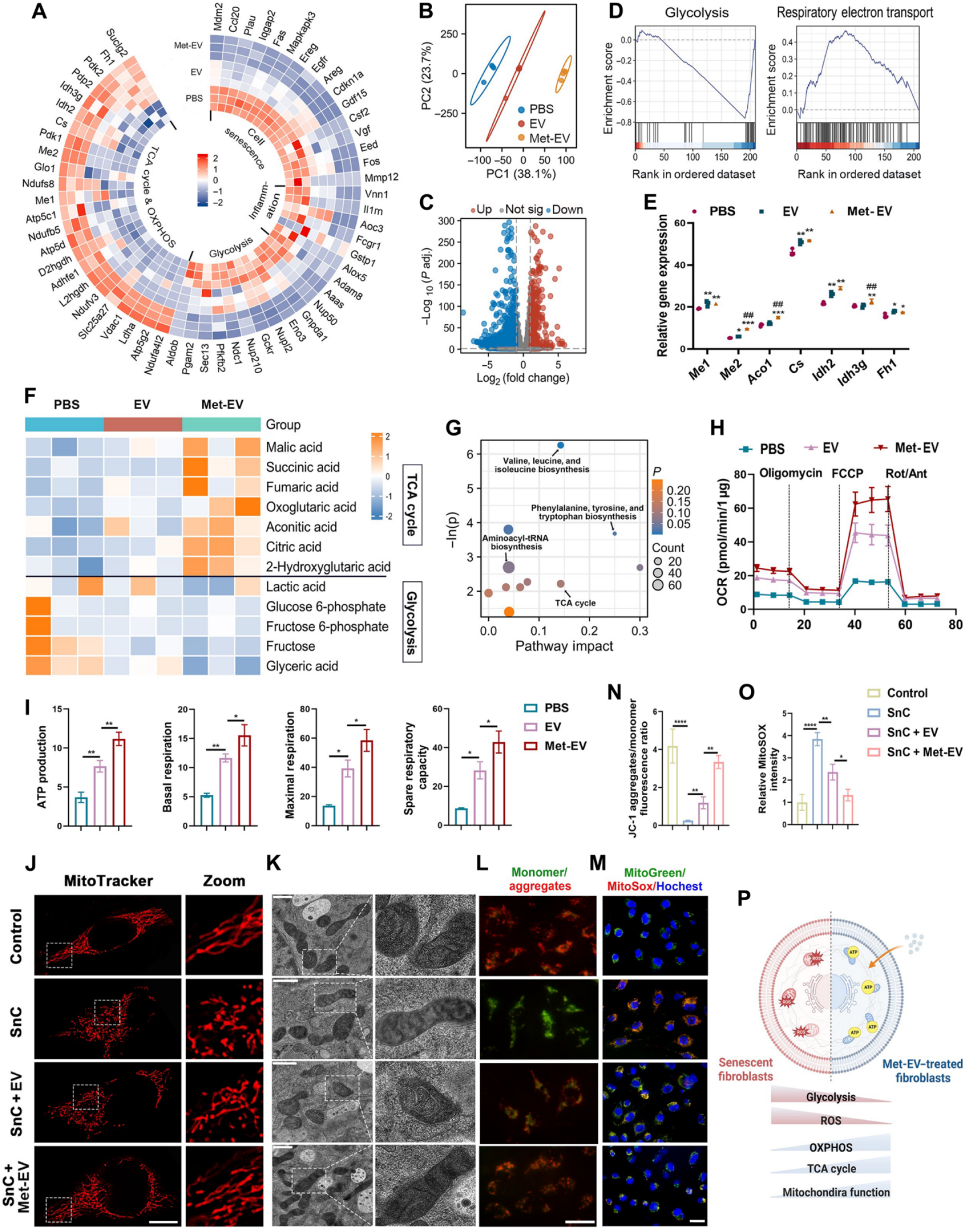
Met-EV stimulate cellular metabolic reprogramming via regulating mitochondria functions. (**A**) Circular heatmap displaying differentially expressed genes related to cell senescence, inflammation, glycolysis, TCA cycle, and OXPHOS, in fibroblasts from aged skin tissues treated with PBS, EV, and Met-EV. (**B**) PCA of genes in PBS, EV, and Met-EV–treated fibroblast groups. (**C**) Volcano plots analysis showed that 40,660 genes were detected, and 7457 genes were identified as differentially expressed genes between PBS and Met-EV groups (absolute log_2_ fold changes > 1 and *P* values < 0.05). (**D**) GSEA was performed to contrast the gene sets associated with glycolysis and OXPHOS between PBS and Met-EV groups. (**E**) Differentially expressed enzymes involved in TCA cycle among PBS, EV, and Met-EV groups. (**F**) Heatmap of differentially detected metabolites in fibroblasts from aged skin tissues treated with PBS, EV, and Met-EV. (**G**) Pathway analysis for the effect of Met-EV on skin tissues compared with PBS using MetaboAnalyst. (**H** and **I**) OCR detection with a Cell Mito Stress Test Kit from Agilent, with ATP production, basal respiration, maximal respiration, and spare respiratory analyzed. (**J**) Representative images of normal and senescent fibroblasts incubated with EV and Met-EV, stained with MitoTracker Red for mitochondria visualization (scale bar: 10 μm). (**K**) Representative TEM images of mitochondria in fibroblasts treated with different stimulus (scale bar: 500 nm). (**L** and **N**) JC-1 aggregates (red) and monomers (green) staining in fibroblasts for detecting mitochondria membrane potential (scale bar: 25 μm) and fluorescence quantitative analysis. (**M** and **O**) MitoGreen for mitochondria tracker and MitoSOX for mtROS detection (scale bar: 30 μm) and fluorescence quantitative analysis. (**P**) Schematic diagram of Met-EV–mediated metabolic reprogramming in senescent fibroblasts. *n* = 3 was analyzed and shown here.

For transcriptomics, results of PCA displayed a completely distinct distribution among PBS, EV, and Met-EV groups (Fig. 6B), and results of volcano plots showed the gene differentiation between PBS and Met-EV groups (Fig. 6C). Figure 6A showed the DEGs among PBS, EV, and Met-EV groups. Expression of genes related to cellular senescence, inflammation, and glycolysis were inhibited, meanwhile TCA cycle and OXPHOS were promoted in EV and Met-EV groups compared with PBS group. KEGG and GO analysis showed that pathways enriched from DEGs were clustered in certain processes like metabolism, wound healing, and collagen deposition. In addition, PI 3-kinase, mitogen-activated protein kinase, adenosine 5′-monophosphate-activated protein kinase, tumor necrosis factor, p53, and hypoxia-inducible factor-1α pathways were also enriched (fig. S6, A and B). The GSEA was consistent with above results, revealing that glycolysis activity was inhibited, while respiratory electron transport was promoted in Met-EV group compared with PBS group (Fig. 6D). The metabolomic results showed that metabolites related to TCA cycle were increased, and metabolites associated with glycolysis were reduced in Met-EV group compared to EV and PBS groups (Fig. 6F). Key enzymes in TCA cycle, which were inhibited in aging skin (Fig. 1L), were rescued with Met-EV treatment (Fig. 6E). Integrative pathway analysis revealed that Met-EV treatment could affect metabolic process like acetyl group transfer into mitochondria, citrate cycle, and Warburg effect (Fig. 6G and fig. S6C). In general, the results indicated that Met-EV treatment drove the metabolic reprogramming, systemically rectifying the imbalance in cellular energy metabolism (glycolysis, TCA cycle, and OXPHOS) around the wound in aging skin.

Mitochondria play an essential role in regulating the metabolic processes, especially governing the TCA cycle and OXPHOS process that happen in mitochondria (*44*). Furthermore, mitochondria dysfunction results in the disturbed oxygen species metabolism, inducing DNA impairment, and accelerating aging process (*45*). Radiation-induced SnCs were used for mitochondrial analysis. To investigate the effect of EV on mitochondria dysfunctions induced by cellular senescence, the morphology, functions, and oxidative metabolic state of mitochondria were analyzed (Fig. 6, H to O).

Oxygen consumption rate (OCR) was performed to characterize OXPHOS process, and the results showed that respiratory capacity–related parameters were reduced after senescence induction and rescued via EV/Met-EV treatment, in which therapeutic effect of Met-EV was stronger than that of EV (Fig. 6, H and I). Mitochondrial morphology of SnCs was discontinuous with obvious fragmentation, while treatment with EV and Met-EV significantly improved the mitochondria impairment, by which morphology was rescued to be continuous and integrate as control cells (Fig. 6J). Moreover, TEM images showed that senescence-induced deficiency in mitochondrial crista and the vagueness of mitochondrial membrane, and EV treatment rescued the mitochondrial morphology (Fig. 6K). Subsequently, oxidative metabolic state was analyzed. Red fluorescence of JC-1 was increased after treatment of EV, which was the indicative of mitochondrial membrane potential restoration and enhanced energy state (Fig. 6, L and N). Mitochondria ROS (mtROS) was increased in SnCs, indicating the oxidative stress damage after senescence induction, and significantly inhibited with EV treatment (Fig. 6, M and O). The flow cytometry analysis also showed that EV, especially Met-EV, reduced the mtROS quantitatively (fig. S6D). Together, the results revealed that Met-EV restored oxidative metabolic balance, morphology, and functions of mitochondria, as well as rectified the imbalance in cellular energy metabolism in aging skin (Fig. 6P).

### Met-EV ameliorate mitochondrial dysfunctions and cell senescence via activating mitophagy

**Fig. 7.**
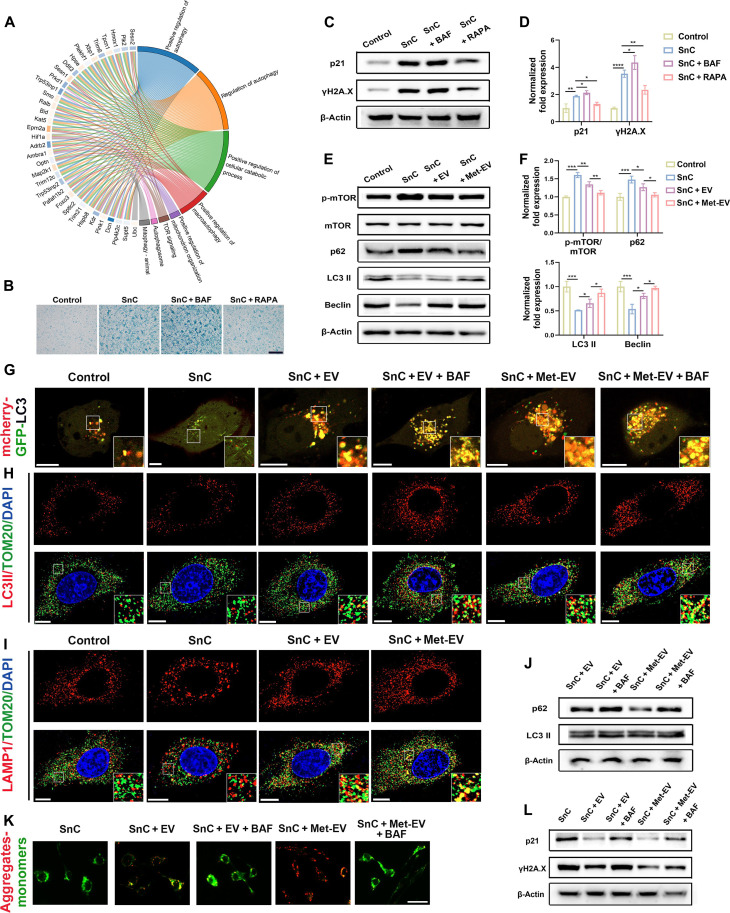
Met-EV ameliorate cell senescence via promoting mitophagy. (**A**) Analysis of functional clustering enrichment for autophagy pathways and relevant DEGs between PBS and Met-EV groups. (**B**) Representative images showing SA-β-Gal in fibroblasts (scale bar: 500 μm). (**C** and **D**) Western blotting results for p16 and γH2A.X expression in fibroblasts treated with rapamycin (RAPA) and bafilomycin A (BAF) and quantitative analysis. (**E** and **F**) Representative immunoblot and quantification for expression of mTOR, p-mTOR, Beclin, P62, and LC3II after 20-hour treatment of EV and Met-EV. (**G**) Representative images of senescent fibroblasts transfected with mCherry-GFP-LC3B adenovirus (scale bars: 10 μm). (**H**) Immunofluorescence images for TOM20 and LC3II after coculture with EV and 4 hours of treatment with BAF (scale bars: 10 μm). (**I**) Immunofluorescence images for TOM20 and LAMP1 (scale bars: 10 μm). (**J**) Representative immunoblot for expression of P62 and LC3II after coculture with EV and 4-hour treatment of BAF. SnCs were exposed to BAF for 4 hours and treated with EV or Met-EV, (**K**) showing the mitochondria membrane potential (scale bar: 30 μm) and (**L**) displaying the immunoblot for p16 and γH2A.X expression. *n* = 3 was analyzed and shown here. TOM20 was used to indicate mitochondria.

Disturbed energy metabolism was reprogrammed, and mitochondria dysfunction was restored in Met-EV–treated fibroblast, but the underlying mechanism for restoration of mitochondria functions requires further exploration. Autophagy, the process for degrading dysfunctional cell components, serves an important role in regulating mitochondrial quality control. Through clearing damaged mitochondria, mitophagy process is indispensable in maintaining mitochondria functions. To identify the role of mitophagy in mitigating cellular senescence and mitochondria dysfunctions, analysis of functional clustering enrichment for autophagy pathways and relevant DEGs between PBS and Met-EV groups was performed. The results showed that autophagy-related DEGs and pathways were enriched, indicating the association between Met-EV treatment and autophagy (Fig. 7A). Furthermore, autophagy agonist could alleviate cellular senescence while antagonist could aggravate senescence, as shown by SA-β-Gal staining (Fig. 7B) and protein expression (Fig. 7, C and D), which further verifying the influence of autophagy on cellular senescence.

Here, senescent fibroblasts induced by radiation were used for autophagy analysis. We found that expression of microtubule-associated protein 1 light chain 3 (LC3 II) and Beclin1 at protein levels was reduced in SnCs while rescued by incubation with Met-EV, and expression of mammalian target of rapamycin (mTOR) and p62 was opposite, revealing that autophagy was suppressed in SnCs and activated with EV especially Met-EV treatment (Fig. 7, E and F). More autophagosomes were observed in Met-EV–treated senescent fibroblasts (fig. S7A). Moreover, colocalization of translocase of outer mitochondrial membrane 20 (TOM20) (indicating mitochondria) and lysosomal associated membrane protein 1 (LAMP1) (indicating lysosome) was suppressed in senescent fibroblasts and increased after Met-EV treatment (Fig. 7I and fig. S7B). These results indicated the deficiency of mitophagy in SnCs and the significantly positive influence of Met-EV on mitophagy.

To further detect patency of autophagic flux and determine the role of autophagy on cellular senescence, bafilomycin A (BAF), an inhibitor of lysosomal acidification, was used to block autophagic flux. Senescent fibroblasts transfected with mCherry-GFP-LC3B adenovirus showed dispersed yellow, indicating the inhibited autophagic flux. Distribution of LC3 puncta showed increased red dots (mCherry^+^/GFP^−^, autolysosomes) and yellow dots (mCherry^+^/GFP^+^, autophagosomes) in Met-EV and EV groups and further increased yellow dots and decreased red dots after BAF treatment, indicating the activated autophagic flux with Met-EV and EV treatment (Fig. 7G). Moreover, colocalization of TOM20 and LC3 II was enhanced in EV and especially in Met-EV group, compared to PBS group, but further increased with treatment of BAF (Fig. 7H and fig. S7C), the results suggesting that Met-EV promoted LC3 II expression via activating autophagy but not blocking autophagosome-lysosome fusion. In addition, protein expression of LC3 II and p62 was also enhanced after BAF treatment (Fig. 7J and fig. S7D), indicating the blockage of autophagic flux via BAF and the accumulated LC3 II after inhibition of autophagic flux. The influence of autophagic flux blockade on cellular senescence and mitochondrial function was then investigated. After autophagy inhibition via BAF, SA-β-Gal activity (fig. S7F) and expression of senescence markers p16 and γH2A.X (Fig. 7L and fig. S7E) were increased, and mitochondria membrane potential was disturbed (Fig. 7K), indicating that Met-EV–mediated mitigation of cellular senescence was dependent, at least in part, on activation of autophagy.

## DISCUSSION

**Fig. 8.**
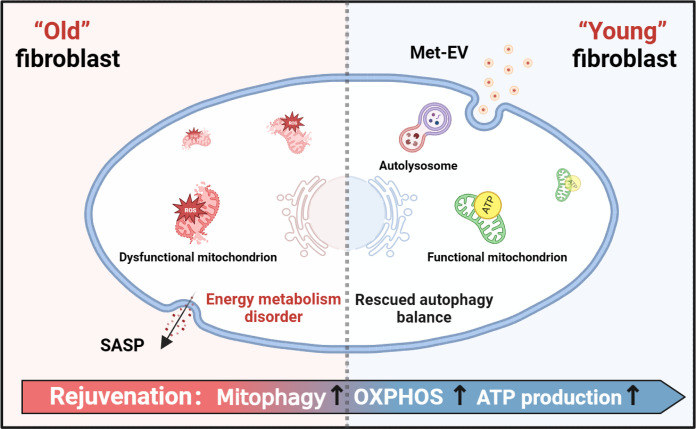
Schematic diagram shows the Met-EV–mediated biological performance. Met-EV could mediate the activation of mitophagy, rescue of mitochondria, and reprogramming of energy metabolism in fibroblasts.

Here, we found that OXPHOS process including TCA cycle and respiratory electron transport was inhibited in aging skin, exhibiting the energy metabolic changes with aging. Then, our data show that metformin improved yields of EV, at the same time Met-EV could significantly promote aged tissue repair, as shown by enhanced skin healing in aging mice in vivo, as well as ameliorated cellular senescence and restored cell dysfunctions in vitro. In particular, ATP metabolism was remodeled as reduced glycolysis and enhanced OXPHOS after Met-EV treatment. Furthermore, senescence-induced accumulation of dysfunctional mitochondria and suppression of mitophagy were rescued via Met-EV, indicating the role of Met-EV in remodeling mitochondrial functions via mitophagy for adequate ATP production in aged tissue repair (Fig. 8). In this regard, our results document that disturbed energy metabolism is essential in age-related defects, which can be a potential therapeutic target for facilitating aged tissue repair.

Aging, the maximum risk factor for many diseases, has close association with progressive function defects at cellular, tissue, and organismal levels (*46*, *47*). Understanding aging and aging-related disease has advanced notably over the past decades, and cellular senescence is considered an essential process in driving age-related declines. Aging-induced cellular senescence can perturb tissue homeostasis via (i) blocking regenerative potential of tissue through proliferative arrest; (ii) changing functions and metabolic states of neighboring cells via secretion of SASP. It has been reported that both the senescence arrest and SASP secretion are associated with cell metabolic conditions (*18*). Age-related declines in the body not only affect longevity but also attenuate capacity of tissue repair.

During aging, the process of metabolism, especially energy metabolism, is important in tissue homeostasis and regeneration. Strategies for regulating cellular metabolism include using vesicles from thylakoid, small molecules (like NAD^+^, peroxisome proliferator-activated receptor γ coactivator 1-α, urolithin A, and spermidine), MSCs, etc. Recently, vesicles from thylakoid have been reported to regulate the ECM anabolism via modulating ATP metabolism, in which more mechanism research is required (*20*). Some small molecules are also reported to be capable of rescuing mitochondrial biogenesis, activating the intracellular antioxidant system, and activating mitophagy to regulate cellular metabolism, but direct administration of small molecules tends to lead to low bioavailability (*12*). Moreover, application of MSCs can regulate biogenesis and improve metabolism, but MSC therapy has inevitable immunogenicity (*12*). EV therapies have low immunogenicity and have been widely applied in extending life span and health span.

It has been known for nearly 20 years that parabiosis are beneficial for the ager via heterochronic blood exchange, with improvement of aging-related parameters (*48*–*50*). Blood from the young are also reported to improve the cognitive function and synaptic plasticity in mice affected by aging (*51*). Since changed microenvironment is one of the ultimate culprits in aging, intercellular communication media EV in blood has been considered as prospective agents for confronting aging in parabiosis (*49*, *52*, *53*). In addition, young cerebrospinal fluid (*54*) and human umbilical cord plasma proteins (*55*) are reported to have potential for revitalizing hippocampal function and restoring memory in mice. It has been shown that EV derived from young cells are capable of alleviating cell senescence as well as promoting health and longevity (*56*–*58*). Moreover, EV derived from MSCs are proven to have intrinsic regenerative capacity, and the EV also has potential for promoting tissue repair in aging mice. Young serum was used for aged skeletal muscle regeneration, and it was shown that circulating EV in serum played an indispensable role (*39*). Young EV derived from human exfoliated deciduous teeth was also found to be able to restore age-related dysfunction of tendon stem cells (*59*). Furthermore, human embryonic stem cell–derived EV rejuvenated senescent bone marrow–derived MSC (BMSC) and ameliorated age-related bone loss (*37*). Similarly, EV from umbilical cord MSC mitigated senescence of BMSC and slowed down age-related bone degeneration via proliferating cell nuclear antigen deposition (*60*).

However, the role of EV in targeting energy metabolism, which plays a key role in aged tissue repair, is limited. Thus, we intended to construct an engineered EV for specific treatment. Considering the ideal therapeutic potential of metformin in mitigating cellular senescence, normalizing mitochondrial functions, and extending life span and its low bioavailability in direct drug delivery, engineering EV with metformin is a potential strategy for combining the advantages of both. Then, we investigated the effect of metformin on MSCs and the therapeutic prospect of Met-EV.

Consistent with the previous studies, we document here that EV from human umbilical cord MSC promoted skin repair in aging mice in vivo (Fig. 5), as well as ameliorated cellular senescence and restored cell dysfunctions in vitro (Figs. 3 and 4). In vitro assays showed that concentration of EV at 10^10^ particles/ml exhibited the best therapeutic effect in inhibiting SA-β-Gal activity and decreasing expression of senescence markers (Fig. 3, B and C). Optimum concentration range for EV functioning might account for the phenomenon that 10^10^ particles/ml EV was more efficient than 10^11^ EV. EV is composed of proteins, RNAs, lipid, etc., and these different composites in EV can play distinct roles in regulating biological behavior. Some molecules in EV can exert positive effects on cell, while some can cause adverse effects, thus there exists an optimum concentration range, the result of which was consistent with previous reports (*61*, *62*).

Metabolic process plays an essential role of in age-related decline (*13*, *14*). Balanced energy metabolism is indispensable for cell activation and functioning in aged tissue repair, but the metabolic shift in SnCs can result in poor ATP production for intracellular anabolism (*19*, *20*). As the direct connection between energy metabolism and aging skin remained to be elucidated, we performed transcriptomics and metabolomics based on human and mouse skin tissues, and results showed that OXPHOS process including TCA cycle and respiratory electron transport was inhibited in aging skin (Fig. 1), indicating that disturbed ATP metabolism can be a potential target for restoring the capacity in aged tissue repair.

As the effect of EV in promoting regeneration in aged tissues and restoring dysfunctions of mitochondria (where OXPHOS takes place), we subsequently explored whether EV treatment rescued cellular senescence and cell functions via reprogramming and balancing energy metabolism. The integrated transcriptomic and metabolomic profiling showed the inhibited glycolysis and increased respiratory electron transport in fibroblasts from aged skin treated with Met-EV (Fig. 6, A to I), indicating a balanced energy metabolism induced by Met-EV treatment. Multiple stimuli can perturb metabolic process, for example, inflammation can increase glycolysis and reduce OXPHOS in chondrocytes, and reprogramming the metabolic state is important for inflammatory modulation (*20*, *63*). Specifically, OXPHOS process is crucial in rescuing regenerative failure of aged stem cells. It was reported that aged satellite cells was activated, and cell regenerative competence was restored via OXPHOS and mitophagy for physiological-aged muscle regeneration (*64*). Apart from EV, platelets were reported to enhance angiogenesis of MSCs via stimulating TCA cycle and activating citrate-dependent fatty acid anabolism (*44*). Above studies and results suggested that restoring imbalanced energy metabolism is crucial in aged tissue repair.

Mitochondria play an important role in regulating age-related declines. Apart from being the place for ATP production, mitochondrial DNA mutations accumulate at an accelerated rate with aging (*65*), and increased expression of catalase target mitochondria can reserve mitochondrial function and extend health span (*66*). Thus, it is known that mitochondria dysfunction can induce cell senescence and SASP secretion. Given this background, we investigated the mitochondria functions with EV treatment and found that EV especially Met-EV rescued senescence-induced mitochondria dysfunctions, characterized as a low membrane potential and a high level of ROS (Fig. 6, J to O), which was favorable for energy metabolic remodeling and SASP inhibition. It has also been reported that restoration of mitochondria dysfunctions is important in ameliorating regenerative failure of MSCs induced by inflammation or diabetes, which is consistent with our results (*67*, *68*).

We further found the activation of autophagy-related DEGs and pathways in Met-EV treatment (Fig. 7A). Actually, mounts of evidence have verified the essential role of autophagy in aging (*45*, *69*), which is also confirmed in Fig. 7 (B and C). Furthermore, mitophagy has attracted much attention in activating cell functions and rescuing mitochondrial dysfunctions (*64*, *68*, *69*). Here, our data showed that Met-EV stimulated the mitophagy and verified the direct effect of mitophagy on senescence (Fig. 7, E to L), which indicated that mitophagy may be an important reason accounting for the restoration of mitochondria dysfunction and mitigation of cellular senescence by Met-EV.

We focus on mitigation of cell dysfunctions and inhibition of SASPs here to confront age-related declines, which is indicated senomorphics. Actually, senolytics, characterized as elimination of SnCs, has also attracted much attention in recent years, proposed as prospects in advancing strategies for age-related pathologies. Appropriate strategies should be selected according to the specific therapeutic requirements.

There are some limitations in this study. Similar to many investigations, the limited number of animals in experimental groups is a challenge to be overcome. EV, especially Met-EV, were proven to be capable of rejuvenating senescent fibroblasts and ECs and improving aged skin repair. However, understanding the molecular changes in EV with metformin engineering and how Met-EV enter and regulate the fate of SnCs in aged tissue repair is necessary. Here, we demonstrated the role of energy metabolism disorders in aging skin and the therapeutic effect of Met-EV in reprogramming the disturbed metabolism, yet it remains unclear how the specific molecules perform a role in modulating metabolic pathways and rejuvenating SnCs. The molecular fate of energy metabolic changes inside recipient SnCs and the functioning bioactive factors (like RNAs, proteins, organelles, etc.) in Met-EV–induced SnC rejuvenation requires further study.

## MATERIALS AND METHODS

### Study design

The objective of the study was to clarify the energy metabolism as a potential target for aged tissue repair and the therapeutic role of Met-EV in regulating metabolic process and rejuvenating SnCs. We firstly conducted transcriptomic and metabolomic analysis with young and aged skin tissues from humans and mice to determine the characteristics of metabolic changes in aging skin. Then, we assessed the role of Met-EV in ameliorating SASP secretion and rejuvenating senescent fibroblasts and ECs at the physiological and genetic levels. We next investigated the therapeutic effects of EV- and Met-EV–loaded hydrogels on skin repair in aging mice (23 to 24 months old) and evaluated epithelization, dermogenesis, and vessel formation. Mice were randomly divided into groups, and experiments were conducted in a double-blinded way. To further determine the molecular mechanisms, we then conducted transcriptomic and metabolomic sequence with fibroblasts from new-born skin tissues in animal experiments and evaluated cell OCR. Mitochondrial morphology, membrane potential, and oxidative stress were also evaluated. Last, to investigate how mitochondria dysfunctions were ameliorated, through inhibition of autophagy flux, mCherry-GFP-LC3 reporter system, Western blotting, and immunofluorescence, we investigated the role of mitophagy in regulating mitochondria dysfunctions and cellular senescence. Detailed experimental methods are available in the Supplementary Materials.

### Mice

The male mice C57BL/6 (23 to 24 months old and 2 months old) were used for the wound healing assays. Five-matched male mice aged 24 to 25 months served as old group, and aged 2 to 3 months served as young group. All the experiments have been proved by the Ethics Committee of Shanghai Ninth People’s Hospital.

### Human samples

The skin tissue samples from five young/adult (16 ± 4 years old) and five aged (77 ± 12 years old) participants were obtained from the Ninth People’s Hospital of the Shanghai Jiao Tong University School of Medicine, with approval from Research Ethics Board in conformity to the Declaration of Helsinki. Human samples were collected with informed consent from patients. When the surgical incisions were trimmed for satisfactory wound suture, the waste skin tissues were collected, rapidly frozen in liquid nitrogen, and stored at −80°C.

### Cell isolation and culture

The human umbilical cord tissues were collected for primary MSC isolation based on previous reports (*70*). Briefly, tissues were rinsed, divided into pieces, attached to the substrate of culture dish (NEST Biotechnology), and cultured in Ham’s F-12K (Kaighn’s) medium (Gibco, 21127022) supplemented with 10% fetal bovine serum (FBS; Cyagen Biosciences, Guangzhou, China, FBSSR-01021-500) and 1% penicillin-streptomycin (Pricella Life Science & Technology, PB180120), at 37°C and 5% CO_2_.

Human umbilical vein endothelial cells [American Type Culture Collection (ATCC), PCS-100-010] and mouse fibroblasts (L cell, ATCC, CRL-2648) were cultured in Dulbecco’s modified Eagle’s medium (DMEM; Gibco, 11965118) supplemented with 10% FBS, at 37°C and 5% CO_2_. Cells were exposed to 10-Gy x-rays and analyzed 10 days later to induce cellular senescence, as previously reported (*71*–*74*).

### Animal procedures

Mice were inhalation-anesthetized with isoflurane, followed by a full-thickness (10 mm in diameter) skin wound made with removing epidermis, dermis, and panniculus carnosus. Aged mice were randomly divided into groups including SA hydrogel + PBS (named PBS), SA hydrogel + EV (named EV), and SA hydrogel + Met-EV (named Met-EV) (*n* = 8). The hydrogel was applied on the wound beds and changed every 6 days. On days 3, 6, 9, and 12 after surgery, wound areas were photographed, and on day 12, skin tissues were collected after hair removal. Wound areas (%) = [area (day *n*)]/[area (day 0)] × 100%, which were calculated via Fiji software. Investigators were blinded to the group division during the period of experiments and analysis.

### Fibroblast harvest

Dermal fibroblasts were isolated from collected skin samples in wound healing assay, according to previous reports (*9*, *75*). After removing subcutaneous fat tissues with a scalpel, skin tissues were incubated in Elastase (0.12 mg/ml; STEMCELL, #07453) in DMEM at 37°C for 25 min to separate dermal and epidermal. Dermis was then diced into pieces and incubated in collagenase IV (4 mg/ml; Gibco, 17104019) in DMEM at 37°C for 1 hour. Digestion process was stopped via DMEM supplemented with 10% FBS. Cell suspension was then filtered with 70-mm cell strainer and pelleted with 1200-rpm centrifugation. Cell pellet was then resuspended in PBS for washing and collected via centrifugation. The supernatant was discarded, and cells were seeded into 75-cm^2^ cell culture flasks (SORFA) in DMEM (Gibco, 11965118) supplemented with 10% FBS, 1% penicillin-streptomycin, and 1% glutamine at 37°C and 5% CO_2_. The obtained fibroblasts were analyzed by gene expression, metabolite distribution, and oxygen consumption.

### Cellular metabolic analysis

Cell OCR was analyzed via Seahorse XFe96 Analyzer. Cells were seed into an XF96-well plate overnight, and cartridge was submerged in distillation-distillation H_2_O at 37°C. On the day of assay, cell culture plate was replaced with detection medium [DMEM supplemented with 1 mM pyruvate, 2 mM glutamine, and 10 mM glucose (pH 7.4)] and incubated in a CO_2_-free incubator for 60 min. Cartridge submerged in prewarmed XF calibrant (Agilent, 100840) was loaded with drugs, by which cells were sequentially treated with 1.5 μM oligomycin, 1 μM carbonyl cyanide *p*-trifluoromethoxyphenylhydrazone, and 1 μM rotenone/antimycin A for Seahorse program analysis. Total protein in each well was analyzed by bicinchoninic acid (Thermo Fisher Scientific, 23227) for normalization.

### Mitochondrial morphology analysis

Cells were incubated with MitoTracker Red CMXRos (50 nM, Invitrogen, M7512) for 15 min at 37°C, then washed with warm PBS, and subsequently observed and photographed using a laser scanning confocal microscope (ZEISS, Germany).

### Autophagy activation and inhibition

To explore the relationship between autophagy and cellular senescence, SnCs were cocultured with rapamycin (RAPA; 100 nM; MedChemExpress, HY-10219) and BAF (10 nM; Sigma-Aldrich, B1793) for 72 hours, in which BAF was added only for the first 4 hours, and then fresh medium was replaced.

### Autophagy/mitophagy flux analysis

For mCherry-GFP-LC3B probe, cells transfected with adenovirus were cultured for 48 hours before experiments. For BAF treatment, after incubation with EV or Met-EV for 20 hours, cells were incubated with BAF (10 nM; Sigma-Aldrich, B1793) for 4 hours at 37°C and cultured for another 3.5 hours to be lysed for Western blotting or fixed for immunofluorescence.

### Statistical analysis

Data were analyzed via GraphPad 9.0 software (San Diego, CA) and represented as mean ± SD from a minimum of three independent experiments. Comparisons between independent groups were analyzed using unpaired Student’s *t* test, and comparisons among multiple groups were performed using one-way analysis of variance (ANOVA) with Tukey comparison test. Statistical significance analysis was conducted using Excel or Prism software, and ∗ indicate the significance among groups: *P < 0.05, **P < 0.01, ***P < 0.001, and ****P < 0.0001.

## Supplementary Materials

### This PDF file includes:

Materials and Methods Figs. S1 to S7 Table S1 References
