# Supplementary material for: Energy metabolism as therapeutic target for aged wound repair by engineered extracellular vesicle
Source: Sci Adv. 2024 Apr 12;10(15):eadl0372. doi: 10.1126/sciadv.adl0372 (PMC11014449; doi:10.1126/sciadv.adl0372)
Supplement: Supplementary file 1 — Materials and Methods Figs. S1 to S7 Table S1 References [file sciadv.adl0372_sm.pdf]

Science Advances
AAAS

# Supplementary Materials for

## Energy metabolism as therapeutic target for aged wound repair by engineered extracellular vesicle

Yu Zhuang *et al.*

Corresponding author: Lingyong Jiang, jianglingyong@sjtu.edu.cn; Xudong Wang, xudongwang70@hotmail.com; Kaili Lin, linkaili@sjtu.edu.cn

*Sci. Adv.* **10**, eadl0372 (2024)
DOI: 10.1126/sciadv.adl0372

**This PDF file includes:**

Materials and Methods
Figs. S1 to S7
Table S1
References

## MATERIALS AND METHODS

### Proliferation assays

MSCs were exposed to different concentrations of metformin (Sigma-Aldrich, PHR1084) for 24 and 48 hours, and cell counting kit-8 (CCK-8, Beyotime, C0037) was utilized to examine the viability, with absorbance examined at 450 nm with a BioTek Epoch (BioTek, USA). The viability of senescent cells was determined using Ki67 staining.

### Ultraviolet–visible spectroscopy (UV-vis)

Appropriate amounts of metformin, EV, EV after sonication, Met-EV and Met-EV after sonication were detected by ultraviolet-spectrophotometer (Implen, Germany), and the ultraviolet absorption curves of different solutions in the wavelength range of 200-800 nm were analyzed.

### Isolation, Characterization, and internalization of the Extracellular Vesicles (EV)

MSCs cultured in exosome-free FBS were treated with 0.5 mM metformin for 48 hours, then the supernatant containing MSCs derived EV was collected. The EV isolation and characterization followed MISEV 2018 guidelines. Cell supernatant was filtered through a 0.22 μm filter (Millipore, SLGP033RB), followed by sequential centrifugation at 2,000 × g for 10 min, 10,000 × g for 30 min, and 100,000 × g for 90 min at 4°C. The EV derived from MSCs were as named as EV, and EV from metformin treated MSCs were named as Met-EV.

For morphology observation, EV loaded on copper grid (carbon-coated) was stained with uranyl acetate, and visualized using TEM. For detection of size distribution, nanoparticle tracking analysis (NTA, NanoSight NS300, Malvern) was utilized. EVs labelled with PKH26 (Sigma, PKH26PCL-1KT) were incubated with cells for internalization assay.

The concentration of $10^{10}$ particles/ml for EV was utilized in this study unless specific described.

**Quantitative proteome analysis**

Samples were analyzed with a nano-UPLC (nanoElute2) coupled to a timsTOF Pro2 instrument (Bruker, Germany) with a nano-electrospray ion source. Then Nano LC-MS/MS analysis was conducted. Vendor's raw MS files were processed using SpectroMine software (4.2.230428.52329) and the built-in Pulsar search engine, for protein identification and quantification.

**Construction and characterization of hydrogel**

For construction of sodium alginate (SA) hydrogel, 1% SA powder and $50\times10^{-3}$ M Ca-EDTA was resolved in $H_2O$ for SA solution, and acetic acid was added for crosslinking to form the porous hydrogel scaffold, followed by washing sequentially with PBS and ethyl alcohol. EV in PBS was resolved in SA solution to construct the EV loaded SA hydrogel ($3\times10^{10}$ particles/scaffold). The SA hydrogel scaffolds were lyophilized for scanning electron microscope (SEM, ZEISS, Germany) observation.

**Histology and Immunofluorescence in skin**

Tissues were fixed with paraformaldehyde (PFA) for 24 hours, then transferred to 70% ethanol, subsequently dehydrated, embedded in paraffin, sliced into sections and deparaffinized for histological analysis. A minimum of 3 mice was analyzed across groups. H&E and Masson staining (Solarbio, G1120 and G1340) were conducted according to manufacturers' instructions.

**Mitochondria membrane potential (ΔΨ) detection**

The mitochondrial potential was analyzed via JC-1 staining. JC-1 probe aggregates in the matrix of the mitochondria to form the polymer at high mitochondrial membrane potential, producing fluorescence at 525 nm; and form the monomer at low potential, producing fluorescence at 490 nm. The JC-1 probe (10 μg/ml, Beyotime, C2006) was co-incubated with cells at 37°C for 20 minutes, and photographed with a ZEISS confocal microscopy. To determine the change in mitochondrial membrane potential, the ratio of red/green fluorescence intensity was analyzed via Fiji software (NIH, USA).

**Intracellular and mitochondrial Reactive oxygen species (ROS) determination**

For detecting the intracellular ROS levels, cells were co-cultured with EV or Mito-TEMPO (20 μM, Sigma-Aldrich, SML0737) after senescence induction. Then, cells were incubated with CellROX Green (5 μM, Invitrogen, C10444) for 20 minutes at 37°C, washed with PBS, and analyzed by fluorescence microscope (ZEISS, Germany) and FACSCalibur Flow Cytometer (BD, USA) at 488nm.

To examine the mitochondrial superoxide, cells were incubated with MitoSOX Red mitochondrial superoxide indicator (5 μM, Invitrogen, M36008) and MitoTracker Green FM (50 nM, Invitrogen, M7514) for 15 minutes at 37°C, and observed using a fluorescence microscope (ZEISS, Germany). Fibroblasts co-cultured with EV or Met-EV were also stained with MitoSOX Green (5 μM, Invitrogen, M36006), and detected by FACSCalibur Flow Cytometer (BD, USA) at 488nm.

**Transmission electron microscopy (TEM)**

Cells were harvested, pelleted and fixed with 2.5% glutaraldehyde in 0.1 M sodium cacodylate buffer (pH 7.0-7.5) at 4°C. The fixed pellets were dehydrated using a gradient ethanol series (50%, 70%, 90%, and 100%), and subsequently embedded in resin with silicone molds for 48 hours at 65°C. Trimmed blocks were then sectioned into ultra-thin sections (70-90 nm thick) using Ultramicrotome (EM UC7, Leica). Then, sections were stained with lead citrate and uranyl acetate solutions respectively for 5-10 minutes, and observed with a HITACHI HT 7800 electron microscope.

**Senescence-associated β-galactosidase (SA-β-Gal) activity**

For SA-β-Gal staining, the kit (Biovision, K320-250) was used based on manufacturer's instructions. Senescent cells (SnCs) were stained as blue and observed with light microscopy.

**RT-qPCR**

Total RNA was extracted from cells utilizing RNAiso (TaKaRa, 9109), and reversed to complementary DNA (cDNA) using Reverse Transcription Kit (TaKaRa, RR036A). Real-time quantitative PCR was performed on a Light Cycler 96 system (Roche) using TB Green Premix Ex Taq™ (TaKaRa, RR420A) and specific primers. β-Actin was used as housekeeping gene control. The threshold cycle (Ct) values were acquired, and analyzed via $2^{-\Delta\Delta Ct}$ method.

List of Gene primer sequences used in this study: ('m' indicates primers for mouse fibroblasts, and 'H' indicates primers for HUVEC)

Table S1. List of Gene primer sequences

| Gene | Forward primer sequence | Reverse primer sequence |
|---|---|---|
| *m-p21* | GTCAGGCTGGTCTGCCTCCG | CGGTCCCGTGGACAGTGAGCAG |
| *m-p53* | CTCTCCCCCGCAAAAGAAAAA | CGGAACATCTCGAAGCGTTTA |
| *m-iNOS* | GAGCGAGTTGTGGATTGTC | CCAGGAAGTAGGTGAGGG |
| *m-IL-1β* | CTGGTACATCAGCACCTCAC | AGAAACAGTCCAGCCCATAC |
| *m-IL-6* | TGCCTTCTTGGGACTGAT | TTGCCATTGCACAACTCTTT |
| *m-β-Actin* | GGTGGGAATGGGTCAGAAGG | GTTGGCCTTAGGGTTCAGGG |
| *H-p21* | TGTCCGTCAGAACCCATGC | AAAGTCGAAGTTCCATCGCTC |
| *H-p53* | CAGCACATGACGGAGGTTGT | TCATCCAAATACTCCACACGC |
| *H-IL-1β* | GCCAACAAGTGGTATTCTCCA | TGCCGTCTTTCATCACACAG |
| *H-IL-6* | CGCCTTCGGTCCAGTTGC | TGGAATCTTCTCCTGGGGGT |
| *H-β-Actin* | GGACTTCGAGCAAGAGATGG | AGCACTGTGTTGGCGTACAG |
| *m-Fh* | AACGTATGCCAATCCCAGTC | CATCTGCGGCCTTCATTATT |
| *m-Me1* | GTCGTGCATCTCTCACAGAAG | TGAGGGCAGTTGGTTTTATCTTT |
| *m-Me2* | TACCACTCCTTGTACCTTGACC | TCTTGTAACGTAAACGCCATTCC |
| *m-Idh1* | GGTTATGGCTCCCTTGGCAT | CCCTTTCTGGTACATGCGGT |
| *m-Idh3g* | TTCCATCCGCAAAGCTGTCT | GTGCCCTGGCCTCCAATATC |
| *H-FH* | TGCAATAATGAAGGCAGCAG | TGATCCAGTCTGCCATACCA |
| *H-ME1* | CTGCTGACACGGAACCCTC | GATCTCCTGACTGTTGAAGGAAG |
| *H-ME2* | ATATACACCGACGGTTGGTCT | CATCAGTCACTACAACAGCCTT |
| *H-IDH1* | ATATTCTGGGTGGCACGGTC | CCGTCACTTGGTGTGTAGGT |
| *H-IDH3g* | CCAGTGGACTTTGAAGAGGTGC | TTTGTGCGACGGTGGCAGGTTA |

**Enzyme linked immunosorbent assay (ELISA)**

Cell supernatant levels of IL-1β and IL-6 were detected with ELISA (ABclonal, RK00006, RK00008, RK00001, RK00004) following the manufacturer's instructions. Samples in duplicate and standards in different concentrations were added into plates

coated with capture antibody, and then plates were sealed and incubated at 37°C for 2 hours. Then biotinylation antibody was added, and plates were sealed at 37°C for 1 hour. Plates were subsequently supplemented with streptavidin–HRP, and sealed for 30 minutes at 37°C, followed by tetramethylbenzidine (TMB) substrate addition, and sealed for 15-20 minutes at 37°C. Finally, stop solution was added, and absorbance of 450 nm was measured for optical density in a microplate reader (BioTek).

**Western blotting**

Cells were lysed in RIPA buffer (Sangon Biotech, C500005) supplemented with protease and phosphatase inhibitor cocktail (Sigma Aldrich, PPC1010) to resolve total proteins with phosphoproteins protected. Proteins in samples were normalized according to BCA results, separated by SDS-PAGE (GenScript Corporation), and then transferred to nitrocellulose membranes (0.45 μm, Sigma Aldrich, HATF00010) or PVDF membranes (0.2 μm, Sigma Aldrich, ISEQ00010). The membranes were blocked with non-fat milk for 60 minutes at RT and then incubated at 4 °C overnight with primary antibodies at proper dilution (1:1000). Secondary antibodies at appropriate dilution (1:5000) were incubated with membranes for 1 hour after Tris Buffered Saline with Tween (TBST, Solarbio, T1081). ECL (Perkin Elmer, NEL105001EA) reagent was utilized for band visualization, and Fiji software was used for image quantification. β-Actin was used as loading control.

Antibodies used for immunoblotting were shown as following: rabbit monoclonal anti-CD63 (ab134045), anti-p21 (ab188224), anti-iNOS (ab178945), rabbit polyclonal anti-Calnexin (ab22595), anti-LC3B (ab48394), and mouse monoclonal anti-β-actin (ab6276) were purchased from Abcam. Mouse monoclonal anti-Alix (sc-53540), anti-ME1 (1:500, sc-100569), anti-IDH3G (1:500, sc-365489), anti-fumarate hydratase (1:500, sc-100743) was purchased from Santa Cruz Biotechnology. Rabbit monoclonal anti-p21 (#2947), anti-Phospho-Histone H2A.X (#9718), anti-Beclin-1 (#3495), and rabbit polyclonal anti-SQSTM1/p62 (#5114), anti-Phospho-mTOR (#2971), anti-mTOR (#2972) were purchased from Cell Signaling Technology. The secondary antibodies anti-mouse IgG, HRP-linked (#7076) and anti-rabbit IgG, HRP-linked (#7074) were purchase from Cell Signaling Technology.

**Immunofluorescence (IF)**

For immunofluorescence, cells were fixed in 4% PFA for 15 minutes, and tissue sections were treated with boiling citric acid (0.01 M, pH=6) for 10 min and cooled for antigen retrieval. Then cells or sections were permeabilized in 0.1% Triton X-100 for 15-30 minutes, and blocked with 10% FBS for 30-60 minutes at RT. Subsequently, samples were incubated with primary antibodies at 4 °C overnight and secondary antibodies at RT for 1 hour. Cells were then stained with DAPI (Beyotime, C1002) and phalloidine (Invitrogen, A12379), and tissue sections were mounted using DAPI Fluoromount-G (Yeasen, 36308ES11). The immunofluorescence intensity and the co-localization of TOM20- LAMP1 and TOM20-LC3 were analyzed using Fiji.

Antibodies used for immunoblotting were shown as following: rabbit monoclonal anti-CD63 (1:500, ab134045), anti-CD31 (1:2000, ab182981), anti-Cytokeratin 14 (1:3000, ab181595), anti-Ki67 (1:250, ab16667), anti-LAMP1 (1:100, ab208943) and rabbit polyclonal anti-Fibronectin (1:500, ab2413), anti-LC3B (1:200, ab48394) were purchase from Abcam. Mouse monoclonal anti-Tom20 (1:200, sc-17764), anti-Alix (1:500, sc-53540) were purchased from Santa Cruz Biotechnology. Rabbit monoclonal anti-Phospho-Histone H2A.X (1:400, #9718) were purchased from Cell Signaling Technology. Mouse monoclonal anti-CD31 (1:400, 66065-2-Ig) and anti-α-SMA (1:400, 67735-1-Ig) was purchased from Proteintech. Secondary antibodies goat anti-rabbit Alexa Fluor 488 (ab150077), 594 (ab150080), and goat anti-mouse 488 (ab150113), 647 (ab150115), with a dilution at 1:1000, were purchased from Abcam.

**Migration assay**

For evaluating the migration ability of cells, scratch wound healing and transwell assays were utilized. In wound healing assay, the mono-cell layer was scratched with p200 pipette tip, and then washed using PBS to remove floating cells. After 24 hours of co-culture with EV or Met-EV, cells were stained with DAPI, and photographed. The migration rate was determined as $(A1 - A0)/A1 \times 100\%$ (A1 representing initial wound width, A0 representing final width)

In transwell assay, cells in serum-free medium were seeded in upper chamber of Transwell plate (8.0 μm pore size, Corning, 3422), and medium with different EV were

added in lower chamber. After 24 hours of co-culture, cells attached on upper surface were removed, and cells on lower was stained with crystal violet and photographed.

**Tube formation assay**

For tube formation *in vitro*, Matrigel™ (Corning, 356234) was coated as the basement of pre-cool plates, then HUVECs preincubated with EV or Met-EV for 48 hours were seeded into Matrigel-coated plates. After 6 hours of co-culture, cells were stained with Calcein AM (MKBio, MX3012), photographed, and quantified by Fiji.

**Transcriptomics analysis**

For RNA sequencing, 1 μg total RNA per sample was used for RNA-seq libraries preparation using NEBNext UltraTM RNA Library Prep Kit (NEB, USA) following manufacturer's recommendations. The clustering of index-coded samples was conducted with TruSeq PE Cluster Kit v3-cBot-HS (Illumia), followed by the sequencing on an Illumina Novaseq platform and generation of 150 bp paired-end reads. Read number counts were obtained via featureCounts v1.5.0-p3, and FPKM of each gene was calculated. $P<0.05$ was considered as differential expression genes (DEGs). GO and KEGG enrichment analysis of DEGs was conducted on g: Profiler platform (https://biit.cs.ut.ee/gprofiler/gost) (76). Gene set enrichment analysis (GSEA) analysis was utilized for comparing pathways between different groups (77).

**Metabolomic analysis**

The LC-MS evaluation was conducted based on an ultra-performance liquid chromatography coupled to tandem mass spectrometry (UPLC-MS/MS) system (ACQUITY UPLC-Xevo TQ-S, Waters Corp., Milford, MA, USA) for metabolomics analysis. Raw data were then processed, peak integrated, calibrated, and quantified for each metabolite utilizing TMBQ software (v1.0, Metabo-Profile, Shanghai, China) (78). For further characterizing the metabolic pathways and changes, MetaboAnalyst 4.0 (www.metaboanalyst.ca) was used for individual metabolites, pathway and enrichment analysis.

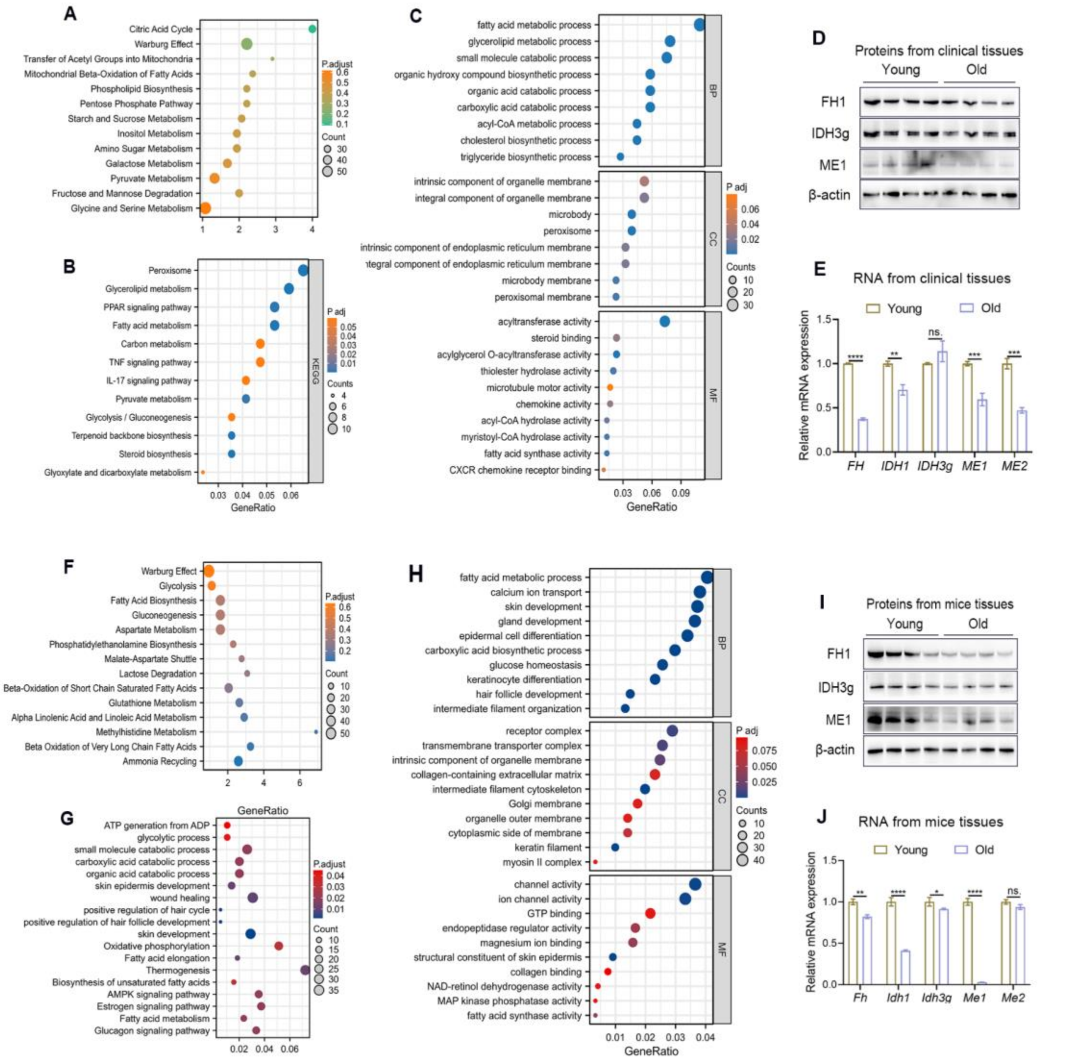


**Fig. S1. Transcriptomics and metabolomics analysis of aging and young skin tissues (related to Fig. 1)**. Enrichment analysis of differentially detected metabolites between young and old groups based on metabolomics in human tissues (A) and mice tissues (F). KEGG enrichment analysis of differentially expressed genes between young and old groups based on transcriptomics in human tissues (B) and mice tissues (G). GO enrichment analysis based on transcriptomics in human tissues (C) and mice tissues (H). Western blot analysis for protein expression in human tissues (D) and mice tissues (I). RT-qPCR analysis for human tissues (E) and mice tissues (J).

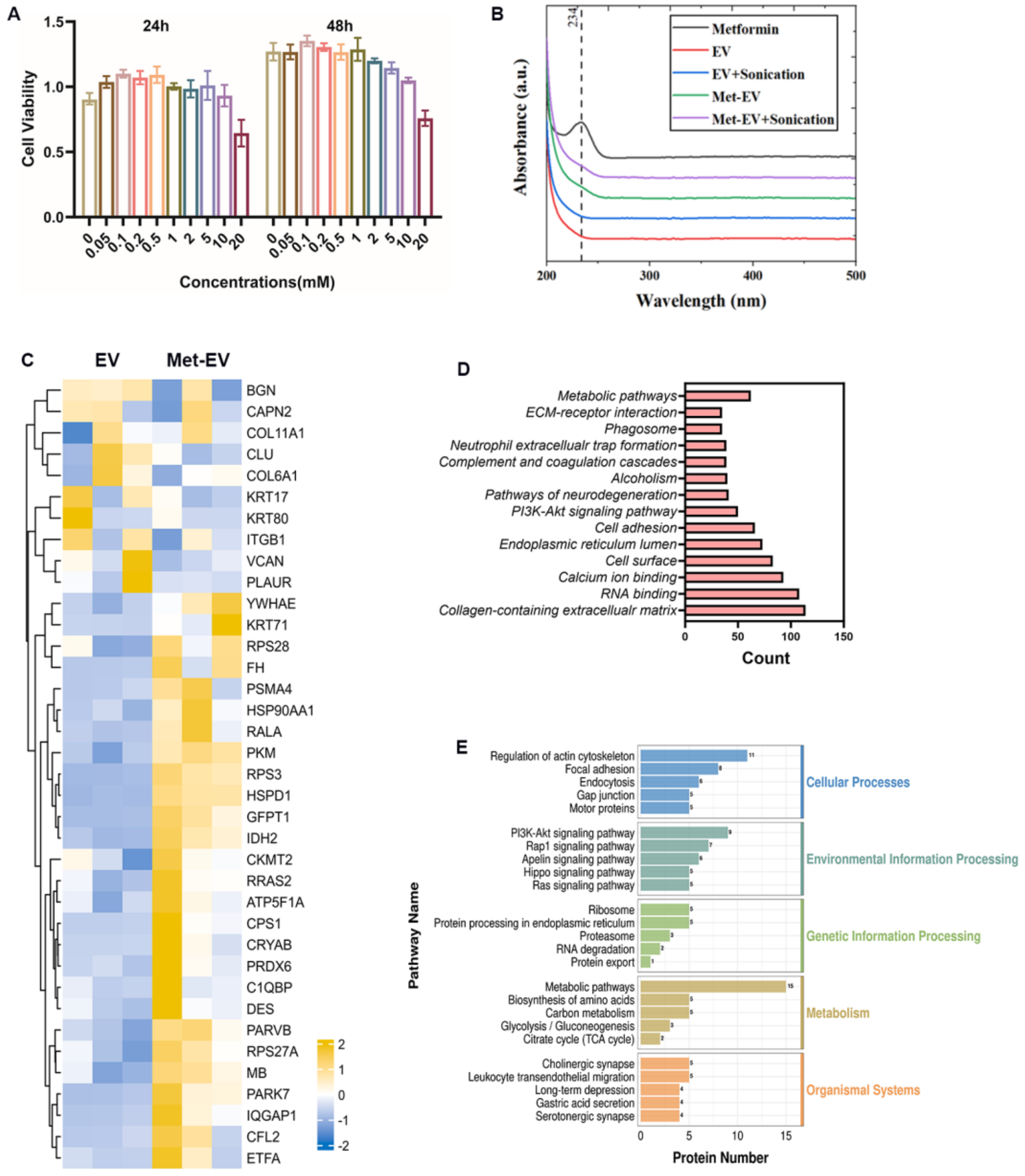


**Fig. S2. Cargos in Met-EV (related to Fig. 2).** (A) Influence of different concentrations of metformin on proliferation of MSCs for 24 h and 48 h via CCK8. n=3 was analyzed and shown here. (B) UV–vis of Metformin, EV, EV after sonication, Met-EV and Met-EV after sonication. (C-E) Quantitative proteome analysis for proteins in EV and Met-EV. (C) Heatmap represented differentially detected proteins in EV and Met-EV groups associated with mitochondrial related metabolism. (D) GO and KEGG functional comments. (E) KEGG enrichment analysis.

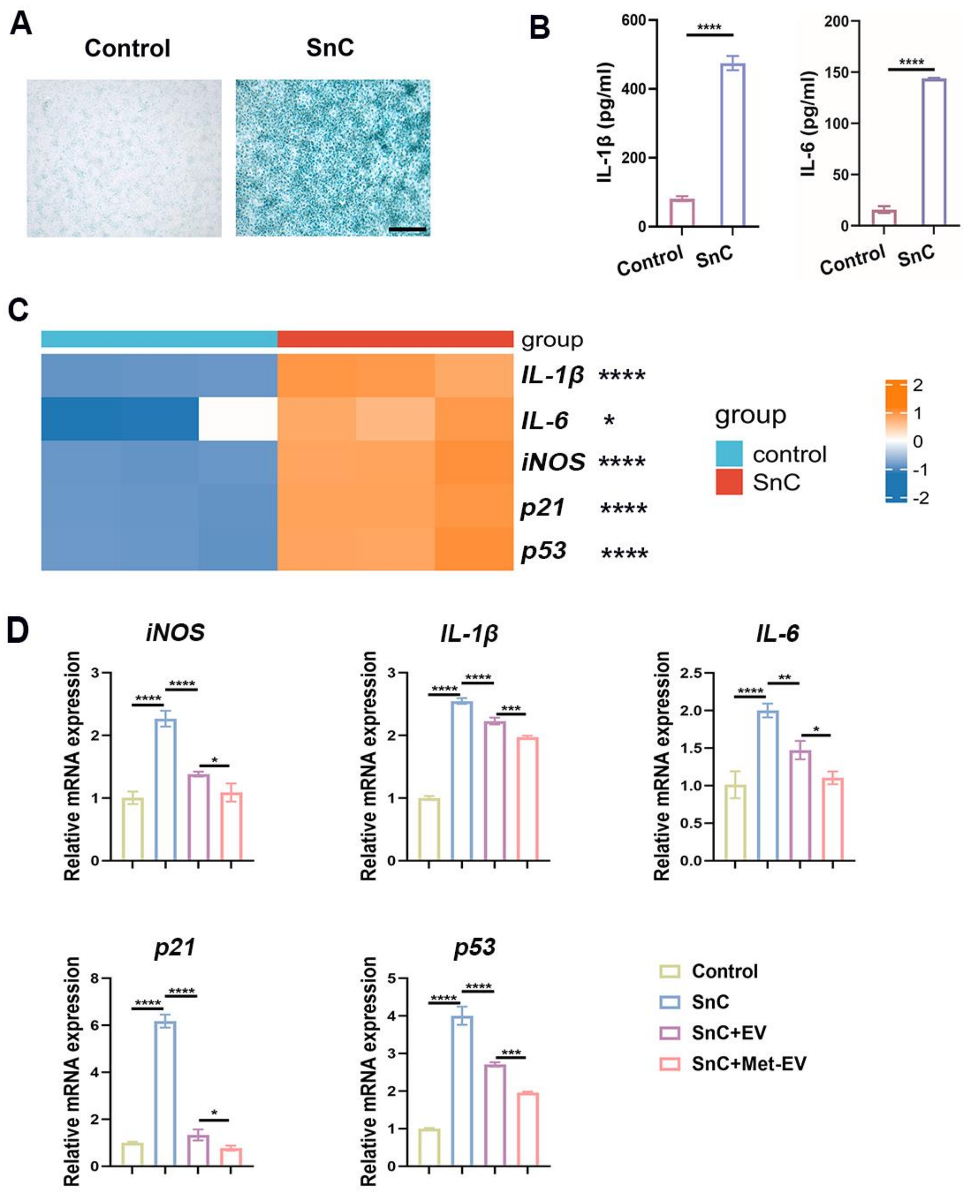


**Fig. S3. Construction of senescent fibroblasts and effect of Met-EV on senescent fibroblasts (related to Fig. 3)**. The senescence phenotype was analyzed 10 days after ionizing radiation. (A) Representative images of SA-β-Gal staining of fibroblasts (scar bar: 500 μm). (B) ELISA analysis for IL-1β, IL-6 secreted from fibroblasts. (C) Heatmap and significance for RT-qPCR analysis of senescence (*p21*, *p53*) and inflammation (*iNOS*, *IL-1β*, *IL-6*) related markers in fibroblasts. (D) Histogram and significance for RT-qPCR analysis of senescence and inflammation related markers in fibroblasts co-cultured with EV for 48 h.

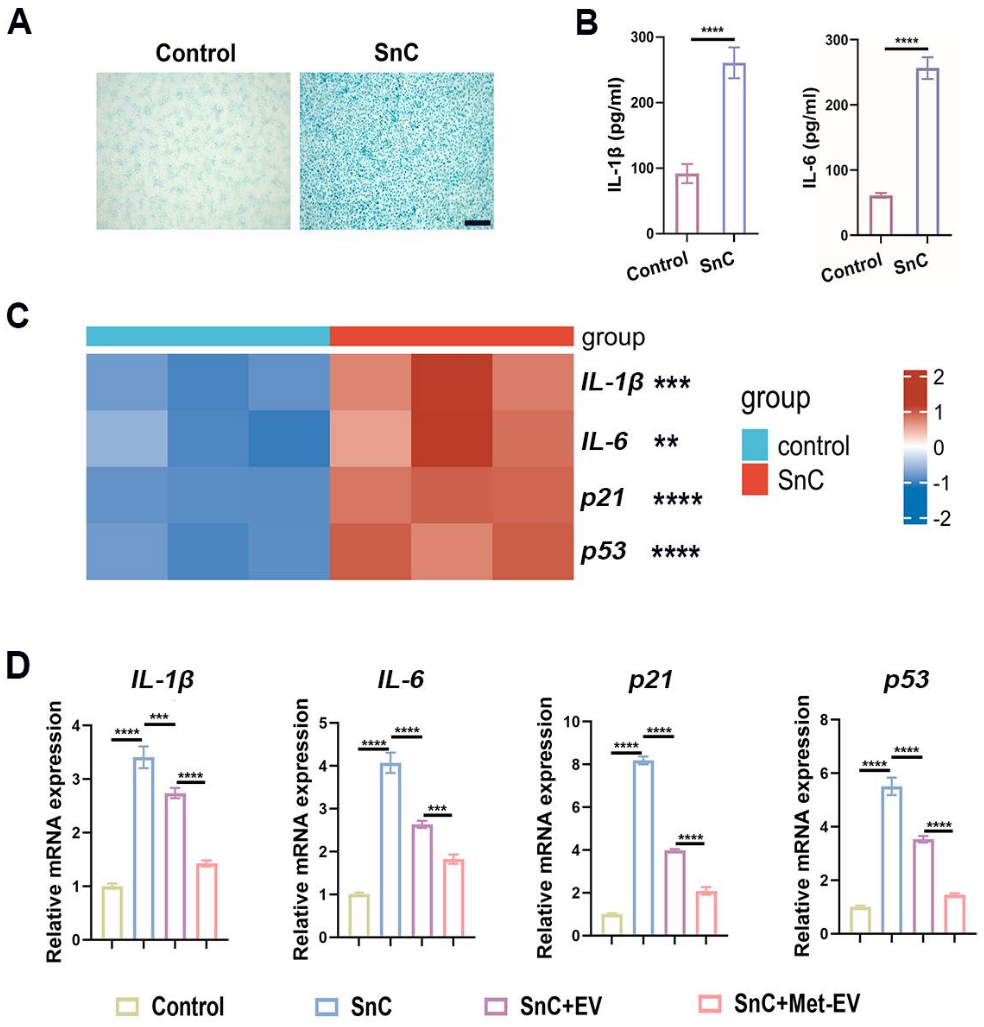


**Fig. S4. Construction of senescent endothelial cells (ECs) and effect of Met-EV on senescent ECs (related to Fig. 4)**. The senescence phenotype was analyzed 10 days after ionizing radiation. (A) Representative images of SA-β-Gal staining of ECs (scar bar: 500 μm). (B) ELISA analysis for IL-1β, IL-6 secreted from ECs. (C) Heatmap and significance for RT-qPCR analysis of senescence (*p21*, *p53*) and inflammation (*IL-1β*, *IL-6*) related markers in ECs. (D) Histogram and significance for RT-qPCR analysis of senescence and inflammation related markers in ECs co-cultured with EV for 48 h.

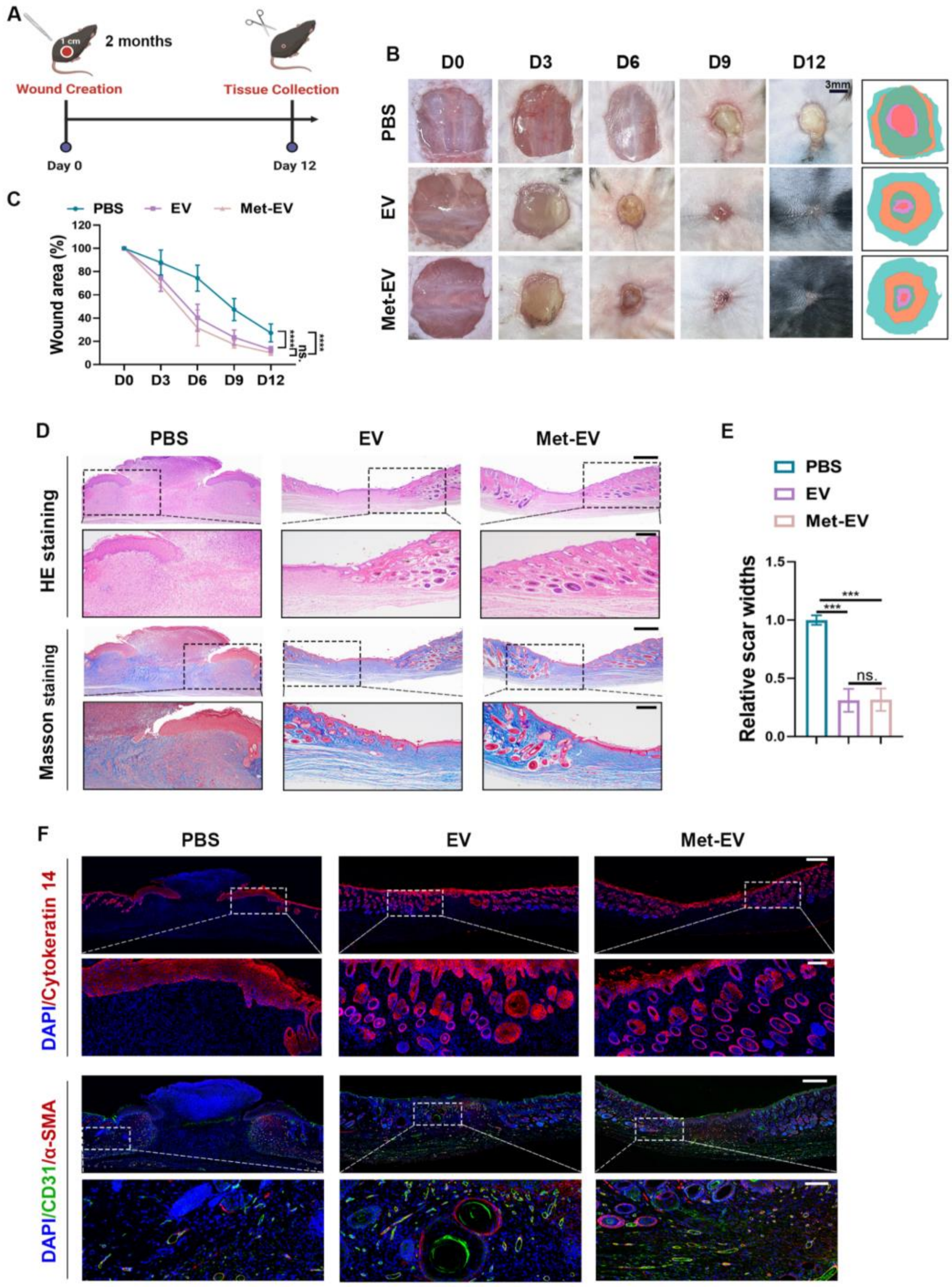


**Fig. S5. Met-EV promote wound healing and blood vessel formation in young mice (related to Fig. 5)**. (A) Schematic illustration of the timeline for skin repair experiments in 2-month mice. (B) Representative wound closure images in young skin at each timepoint after operation treated with EV functionalized hydrogel (scar bar: 3 mm), and corresponding wound area tracing (n=7). (C) Quantitative analysis of remaining wound area (n=7). (D) H&E and Masson's trichrome staining of repaired skin tissues at day 12 (scar bar: 500 μm, 200 μm for magnification), and quantitative analysis of scar widths were shown in (E) (n=3). (F) Representative immunofluorescence images of cytokeratin 14 (red), and fluorescence co-staining of CD31 and α-SMA in newly-born skin tissues (scar bar: 500 μm, 100 μm for magnification) (n=3).

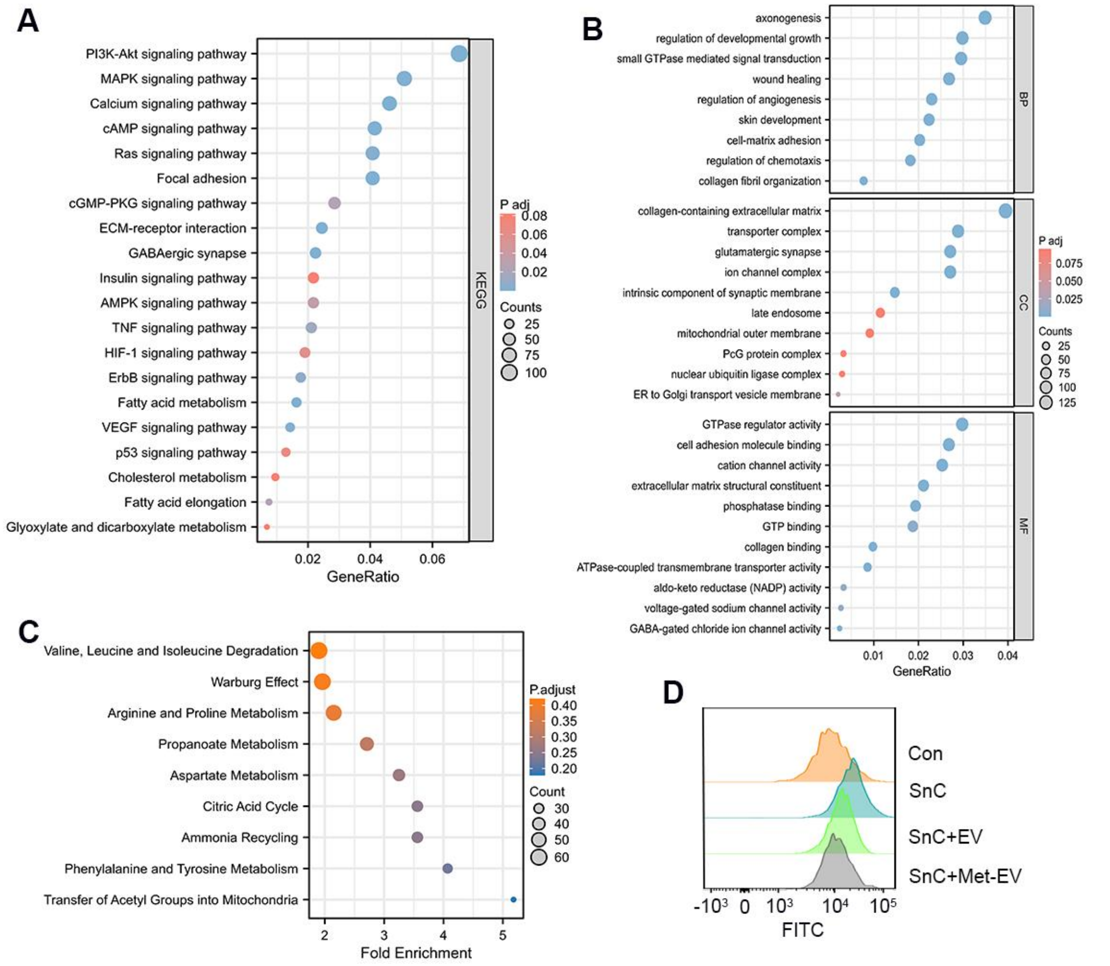


**Fig. S6. Transcriptomics and metabolomics analysis of PBS and Met-EV treat fibroblasts and mtSOX for fibroblasts (related to Fig. 6)**. (A) Enrichment analysis of differentially detected metabolites between PBS and Met-EV groups based on metabolomics. (B) KEGG and (C) GO enrichment analysis of differentially expressed genes between PBS and Met-EV groups based on transcriptomics. (D) Flow cytometry analysis for mtROS detection with MitoSOX staining.

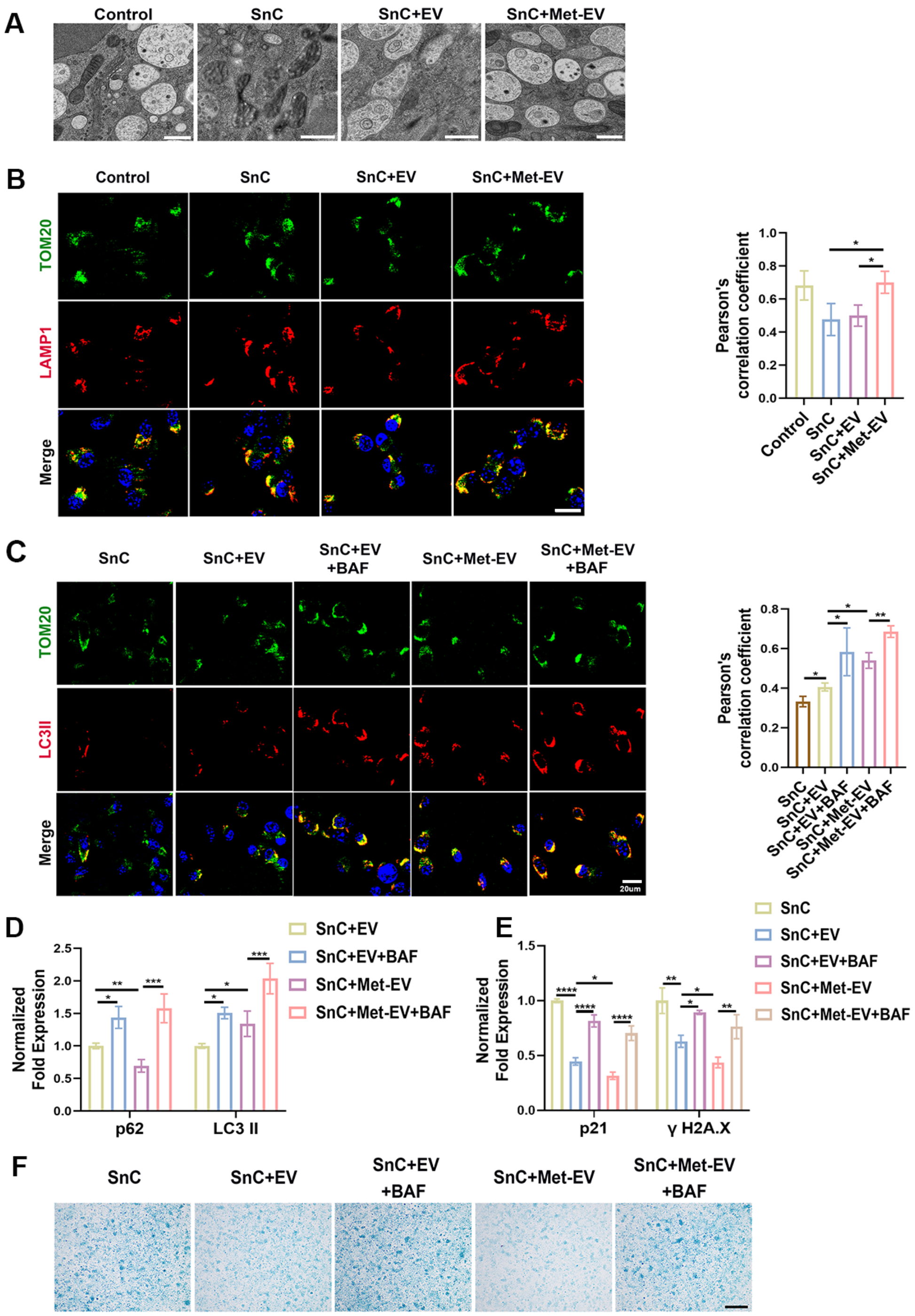


**Fig. S7. Met-EV ameliorates cell senescence via promoting mitophagy (related to Fig. 7)**. (A) Representative TEM images of autophagosome in fibroblasts treated with different stimulus (scar bar: 500 nm). (B) Immunofluorescence images for TOM20 (top) and LAMP1 (middle), and quantification of co-localization of LAMP to mitochondria (right) (scar bar: 20 μm). (C) Immunofluorescence images for TOM20 (top) and LC3II (middle), and quantification of co-localization of LC3 to mitochondria (right) after 4 h of treatment with BAF (scar bar: 20 μm). (D) displaying the quantification for LC3II and p62 expression in the immunoblot. (E) displaying the quantification for p21 and γ H2A.X expression in the immunoblot. (F) showing the SA-β-gal in fibroblasts (scar bar: 500 μm).

**REFERENCE AND NOTES**


1. E. Fuchs, Scratching the surface of skin development. *Nature* **445**, 834–842 (2007).

2. B. E. Keyes, S. Liu, A. Asare, S. Naik, J. Levorse, L. Polak, C. P. Lu, M. Nikolova, H. A. Pasolli, E. Fuchs, Impaired epidermal to dendritic T cell signaling slows wound repair in aged skin. *Cell* **167**, 1323–1338.e14 (2016).

3. J. Oh, Y. D. Lee, A. J. Wagers, Stem cell aging: Mechanisms, regulators and therapeutic opportunities. *Nat. Med.* **20**, 870–880 (2014).

4. B. K. Kennedy, S. L. Berger, A. Brunet, J. Campisi, A. M. Cuervo, E. S. Epel, C. Franceschi, G. J. Lithgow, R. I. Morimoto, J. E. Pessin, T. A. Rando, A. Richardson, E. E. Schadt, T. Wyss-Coray, F. Sierra, Geroscience: Linking aging to chronic disease. *Cell* **159**, 709–713 (2014).

5. M. C. Velarde, M. Demaria, S. Melov, J. Campisi, Pleiotropic age-dependent effects of mitochondrial dysfunction on epidermal stem cells. *Proc. Natl. Acad. Sci. U.S.A.* **112**, 10407–10412 (2015).

6. O. H. Jeon, C. Kim, R.-M. Laberge, M. Demaria, S. Rathod, A. P. Vasserot, J. W. Chung, D. H. Kim, Y. Poon, N. David, D. J. Baker, J. M. van Deursen, J. Campisi, J. H. Elisseeff, Local clearance of senescent cells attenuates the development of post-traumatic osteoarthritis and creates a pro-regenerative environment. *Nat. Med.* **23**, 775–781 (2017).

7. S. He, N. E. Sharpless, Senescence in health and disease. *Cell* **169**, 1000–1011 (2017).

8. R. Di Micco, V. Krizhanovsky, D. Baker, F. d’Adda di Fagagna, Cellular senescence in ageing: From mechanisms to therapeutic opportunities. *Nat. Rev. Mol. Cell Biol.* **22**, 75–95 (2021).

9. T. Hasegawa, T. Oka, H. G. Son, V. S. Oliver-García, M. Azin, T. M. Eisenhaure, D. J. Lieb, N. Hacohen, S. Demehri, Cytotoxic $CD4^+$ T cells eliminate senescent cells by targeting cytomegalovirus antigen. *Cell* **186**, 1417–1431.e20 (2023).

10. S. Mahmoudi, L. Xu, A. Brunet, Turning back time with emerging rejuvenation strategies. *Nat. Cell Biol.* **21**, 32–43 (2019).

11. A. Hernandez-Segura, J. Nehme, M. Demaria, Hallmarks of cellular senescence. *Trends Cell Biol.* **28**, 436–453 (2018).

12. R. Ren, A. Ocampo, G.-H. Liu, J. C. Izpisua Belmonte, Regulation of stem cell aging by metabolism and epigenetics. *Cell Metab.* **26**, 460–474 (2017).

13. N. S. Chandel, H. Jasper, T. T. Ho, E. Passegué, Metabolic regulation of stem cell function in tissue homeostasis and organismal ageing. *Nat. Cell Biol.* **18**, 823–832 (2016).

14. L. García-Prat, P. Sousa-Victor, P. Muñoz-Cánoves, Proteostatic and metabolic control of stemness. *Cell Stem Cell* **20**, 593–608 (2017).

15. E. L. James, R. D. Michalek, G. N. Pitiyage, A. M. de Castro, K. S. Vignola, J. Jones, R. P. Mohney, E. D. Karoly, S. S. Prime, E. K. Parkinson, Senescent human fibroblasts show increased glycolysis and redox homeostasis with extracellular metabolomes that overlap with those of irreparable DNA Damage, aging, and disease. *J. Proteome Res.* **14**, 1854–1871 (2015).

16. A. H. Bittles, N. Harper, Increased glycolysis in ageing cultured human diploid fibroblasts. *Biosci. Rep.* **4**, 751–756 (1984).

17. W. Zwerschke, S. Mazurek, P. Stöckl, E. Hütter, E. Eigenbrodt, P. Jansen-dürr, Metabolic analysis of senescent human fibroblasts reveals a role for AMP in cellular senescence. *Biochem. J.* **376**, 403–411 (2003).

18. C. D. Wiley, J. Campisi, From ancient pathways to aging cells—connecting metabolism and cellular senescence. *Cell Metab.* **23**, 1013–1021 (2016).

19. E. Sahin, R. A. DePinho, Linking functional decline of telomeres, mitochondria and stem cells during ageing. *Nature* **464**, 520–528 (2010).

20. P. Chen, X. Liu, C. Gu, P. Zhong, N. Song, M. Li, Z. Dai, X. Fang, Z. Liu, J. Zhang, R. Tang, S. Fan, X. Lin, A plant-derived natural photosynthetic system for improving cell anabolism. *Nature* **612**, 546–554 (2022).

21. P. Rustin, Mitochondria, from cell death to proliferation. *Nat. Genet.* **30**, 352–353 (2002).

22. D.-W. Zheng, L. Xu, C.-X. Li, X. Dong, P. Pan, Q.-L. Zhang, B. Li, X. Zeng, X.-Z. Zhang, Photo-powered artificial organelles for ATP generation and life-sustainment. *Adv. Mater.* **30**, e1805038 (2018).

23. J. A. Amorim, G. Coppotelli, A. P. Rolo, C. M. Palmeira, J. M. Ross, D. A. Sinclair, Mitochondrial and metabolic dysfunction in ageing and age-related diseases. *Nat. Rev. Endocrinol.* **18**, 243–258 (2022).

24. N. van Gastel, G. Carmeliet, Metabolic regulation of skeletal cell fate and function in physiology and disease. *Nat. Metab.* **3**, 11–20 (2021).

25. C. D. Wiley, J. Campisi, The metabolic roots of senescence: Mechanisms and opportunities for intervention. *Nat. Metab.* **3**, 1290–1301 (2021).

26. Christopher D. Wiley, Michael C. Velarde, P. Lecot, S. Liu, Ethan A. Sarnoski, A. Freund, K. Shirakawa, Hyung W. Lim, Sonnet S. Davis, A. Ramanathan, Akos A. Gerencser, E. Verdin, J. Campisi, Mitochondrial dysfunction induces senescence with a distinct secretory phenotype. *Cell Metab.* **23**, 303–314 (2016).

27. J. P. White, A. N. Billin, M. E. Campbell, A. J. Russell, K. M. Huffman, W. E. Kraus, The AMPK/p27$^{Kip1}$ axis regulates autophagy/apoptosis decisions in aged skeletal muscle stem cells. *Stem Cell Rep.* **11**, 425–439 (2018).

28. L. García-Prat, M. Martínez-Vicente, E. Perdiguero, L. Ortet, J. Rodríguez-Ubreva, E. Rebollo, V. Ruiz-Bonilla, S. Gutarra, E. Ballestar, A. L. Serrano, M. Sandri, P. Muñoz-Cánoves, Autophagy maintains stemness by preventing senescence. *Nature* **529**, 37–42 (2016).

29. A. H. Tang, T. A. Rando, Induction of autophagy supports the bioenergetic demands of quiescent muscle stem cell activation. *EMBO J.* **33**, 2782–2797 (2014).

30. P. Sousa-Victor, L. García-Prat, P. Muñoz-Cánoves, Control of satellite cell function in muscle regeneration and its disruption in ageing. *Nat. Rev. Mol. Cell Biol.* **23**, 204–226 (2022).

31. C. Correia-Melo, F. D. Marques, R. Anderson, G. Hewitt, R. Hewitt, J. Cole, B. M. Carroll, S. Miwa, J. Birch, A. Merz, M. D. Rushton, M. Charles, D. Jurk, S. W. Tait, R. Czapiewski, L. Greaves, G. Nelson, M. Bohlooly-Y, S. Rodriguez-Cuenca, A. Vidal-Puig, D. Mann, G. Saretzki, G. Quarato, D. R. Green, P. D. Adams, T. von Zglinicki, V. I. Korolchuk, J. F. Passos, Mitochondria are required for pro-ageing features of the senescent phenotype. *EMBO J.* **35**, 724–742 (2016).

32. Y. Zhuang, M. Cheng, M. Li, J. Cui, J. Huang, C. Zhang, J. Si, K. Lin, H. Yu, Small extracellular vesicles derived from hypoxic mesenchymal stem cells promote vascularized bone regeneration through the miR-210-3p/EFNA3/PI3K pathway. *Acta Biomater.* **150**, 413–426 (2022).

33. Y. Zhuang, S. Jiang, C. Yuan, K. Lin, The potential therapeutic role of extracellular vesicles in osteoarthritis. *Front. Bioeng. Biotechnol.* **10**, 1022368 (2022).

34. Z. Liu, Y. Zhuang, L. Fang, C. Yuan, X. Wang, K. Lin, Breakthrough of extracellular vesicles in pathogenesis, diagnosis and treatment of osteoarthritis. *Bioact. Mater.* **22**, 423–452 (2023).

35. J. A. Fafián-Labora, J. A. Rodríguez-Navarro, A. O'Loghlen, Small extracellular vesicles have gst activity and ameliorate senescence-related tissue damage. *Cell Metab.* **32**, 71–86.e5 (2020).

36. R. Feng, M. Ullah, K. Chen, Q. Ali, Y. Lin, Z. Sun, Stem cell-derived extracellular vesicles mitigate ageing-associated arterial stiffness and hypertension. *J. Extracell. Vesicles* **9**, 1783869 (2020).

37. L. Gong, B. Chen, J. Zhang, Y. Sun, J. Yuan, X. Niu, G. Hu, Y. Chen, Z. Xie, Z. Deng, Q. Li, Y. Wang, Human ESC-sEVs alleviate age-related bone loss by rejuvenating senescent bone marrow-derived mesenchymal stem cells. *J. Extracell. Vesicles* **9**, 1800971 (2020).

38. X. Xiao, M. Xu, H. Yu, L. Wang, X. Li, J. Rak, S. Wang, R. C. Zhao, Mesenchymal stem cell-derived small extracellular vesicles mitigate oxidative stress-induced senescence in endothelial cells via regulation of miR-146a/Src. *Signal Transduct. Target. Ther.* **6**, 354 (2021).

39. A. Sahu, Z. J. Clemens, S. N. Shinde, S. Sivakumar, A. Pius, A. Bhatia, S. Picciolini, C. Carlomagno, A. Gualerzi, M. Bedoni, B. Van Houten, M. Lovalekar, N. F. Fitz, I. Lefterov, A. Barchowsky, R. Koldamova, F. Ambrosio, Regulation of aged skeletal muscle regeneration by circulating extracellular vesicles. *Nat. Aging* **1**, 1148–1161 (2021).

40. L. Zheng, Y. Wang, P. Qiu, C. Xia, Y. Fang, S. Mei, C. Fang, Y. Shi, K. Wu, Z. Chen, S. Fan, D. He, X. Lin, P. Chen, Primary chondrocyte exosomes mediate osteoarthritis progression by regulating mitochondrion and immune reactivity. *Nanomedicine* **14**, 3193–3212 (2019).

41. P. Chen, L. Zheng, Y. Wang, M. Tao, Z. Xie, C. Xia, C. Gu, J. Chen, P. Qiu, S. Mei, L. Ning, Y. Shi, C. Fang, S. Fan, X. Lin, Desktop-stereolithography 3D printing of a radially oriented extracellular matrix/mesenchymal stem cell exosome bioink for osteochondral defect regeneration. *Theranostics* **9**, 2439–2459 (2019).

42. L. P. Bharath, M. Agrawal, G. McCambridge, D. A. Nicholas, H. Hasturk, J. Liu, K. Jiang, R. Liu, Z. Guo, J. Deeney, C. M. Apovian, J. Snyder-Cappione, G. S. Hawk, R. M. Fleeman, R. M. F. Pihl, K. Thompson, A. C. Belkina, L. Cui, E. A. Proctor, P. A. Kern, B. S. Nikolajczyk, Metformin enhances autophagy and normalizes mitochondrial function to alleviate aging-associated inflammation. *Cell Metab.* **32**, 44–55.e6 (2020).

43. A. S. Kulkarni, S. Gubbi, N. Barzilai, Benefits of metformin in attenuating the hallmarks of aging. *Cell Metab.* **32**, 15–30 (2020).

44. J. Levoux, A. Prola, P. Lafuste, M. Gervais, N. Chevallier, Z. Koumaiha, K. Kefi, L. Braud, A. Schmitt, A. Yacia, A. Schirmann, B. Hersant, M. Sid-Ahmed, S. Ben Larbi, K. Komrskova, J. Rohlena, F. Relaix, J. Neuzil, A.-M. Rodriguez, Platelets facilitate the wound-healing capability of mesenchymal stem cells by mitochondrial transfer and metabolic reprogramming. *Cell Metab.* **33**, 283–299.e9 (2021).

45. F. Liu, Y. Yuan, L. Bai, L. Yuan, L. Li, J. Liu, Y. Chen, Y. Lu, J. Cheng, J. Zhang, LRRc17 controls BMSC senescence via mitophagy and inhibits the therapeutic effect of BMSCs on ovariectomy-induced bone loss. *Redox Biol.* **43**, 101963 (2021).

46. J. Campisi, P. Kapahi, G. J. Lithgow, S. Melov, J. C. Newman, E. Verdin, From discoveries in ageing research to therapeutics for healthy ageing. *Nature* **571**, 183–192 (2019).

47. L. Katsimpardi, N. K. Litterman, P. A. Schein, C. M. Miller, F. S. Loffredo, G. R. Wojtkiewicz, J. W. Chen, R. T. Lee, A. J. Wagers, L. L. Rubin, Vascular and neurogenic rejuvenation of the aging mouse brain by young systemic factors. *Science* **344**, 630–634 (2014).

48. S. Ma, S. Wang, Y. Ye, J. Ren, R. Chen, W. Li, J. Li, L. Zhao, Q. Zhao, G. Sun, Y. Jing, Y. Zuo, M. Xiong, Y. Yang, Q. Wang, J. Lei, S. Sun, X. Long, M. Song, S. Yu, P. Chan, J. Wang, Q. Zhou, J. C. I. Belmonte, J. Qu, W. Zhang, G.-H. Liu, Heterochronic parabiosis induces stem cell revitalization and systemic rejuvenation across aged tissues. *Cell Stem Cell* **29**, 990–1005.e10 (2022).

49. I. M. Conboy, M. J. Conboy, A. J. Wagers, E. R. Girma, I. L. Weissman, T. A. Rando, Rejuvenation of aged progenitor cells by exposure to a young systemic environment. *Nature* **433**, 760–764 (2005).

50. O. H. Jeon, M. Mehdipour, T.-H. Gil, M. Kang, N. W. Aguirre, Z. R. Robinson, C. Kato, J. Etienne, H. G. Lee, F. Alimirah, V. Walavalkar, P.-Y. Desprez, M. J. Conboy, J. Campisi, I. M. Conboy, Systemic induction of senescence in young mice after single heterochronic blood exchange. *Nat. Metab.* **4**, 995–1006 (2022).

51. S. A. Villeda, K. E. Plambeck, J. Middeldorp, J. M. Castellano, K. I. Mosher, J. Luo, L. K. Smith, G. Bieri, K. Lin, D. Berdnik, R. Wabl, J. Udeochu, E. G. Wheatley, B. Zou, D. A. Simmons, X. S. Xie, F. M. Longo, T. Wyss-Coray, Young blood reverses age-related impairments in cognitive function and synaptic plasticity in mice. *Nat. Med.* **20**, 659–663 (2014).

52. F. S. Loffredo, M. L. Steinhauser, S. M. Jay, J. Gannon, J. R. Pancoast, P. Yalamanchi, M. Sinha, C. Dall'Osso, D. Khong, J. L. Shadrach, C. M. Miller, B. S. Singer, A. Stewart, N. Psychogios, R. E. Gerszten, A. J. Hartigan, M.-J. Kim, T. Serwold, A. J. Wagers, R. T. Lee, Growth differentiation factor 11 is a circulating factor that reverses age-related cardiac hypertrophy. *Cell* **153**, 828–839 (2013).

53. P. Sousa-Victor, J. Neves, W. Cedron-Craft, P. B. Ventura, C.-Y. Liao, R. R. Riley, I. Soifer, N. van Bruggen, G. A. Kolumam, S. A. Villeda, D. A. Lamba, H. Jasper, MANF regulates metabolic and immune homeostasis in ageing and protects against liver damage. *Nat. Metab.* **1**, 276–290 (2019).

54. T. Iram, F. Kern, A. Kaur, S. Myneni, A. R. Morningstar, H. Shin, M. A. Garcia, L. Yerra, R. Palovics, A. C. Yang, O. Hahn, N. Lu, S. R. Shuken, M. S. Haney, B. Lehallier, M. Iyer, J. Luo, H. Zetterberg, A. Keller, J. B. Zuchero, T. Wyss-Coray, Young CSF restores oligodendrogenesis and memory in aged mice via Fgf17. *Nature* **605**, 509–515 (2022).

55. J. M. Castellano, K. I. Mosher, R. J. Abbey, A. A. McBride, M. L. James, D. Berdnik, J. C. Shen, B. Zou, X. S. Xie, M. Tingle, I. V. Hinkson, M. S. Angst, T. Wyss-Coray, Human umbilical cord plasma proteins revitalize hippocampal function in aged mice. *Nature* **544**, 488–492 (2017).

56. M. Yoshida, A. Satoh, J. B. Lin, K. F. Mills, Y. Sasaki, N. Rensing, M. Wong, R. S. Apte, S.-I. Imai, Extracellular vesicle-contained eNAMPT delays aging and extends lifespan in mice. *Cell Metab.* **30**, 329–342.e5 (2019).

57. J. Sanz-Ros, N. Romero-García, C. Mas-Bargues, D. Monleón, J. Gordevicius, R. T. Brooke, M. Dromant, A. Díaz, A. Derevyanko, A. Guío-Carrión, A. Román-Domínguez, M. Inglés, M. A. Blasco, S. Horvath, J. Viña, C. Borrás, Small extracellular vesicles from young adipose-derived stem cells prevent frailty, improve health span, and decrease epigenetic age in old mice. *Sci. Adv.* **8**, eabq2226 (2022).

58. F. Prattichizzo, A. Giuliani, J. Sabbatinelli, E. Mensà, V. De Nigris, L. La Sala, P. de Candia, F. Olivieri, A. Ceriello, Extracellular vesicles circulating in young organisms promote healthy longevity. *J. Extracell. Vesicles* **8**, 1656044 (2019).

59. S. Jin, Y. Wang, X. Wu, Z. Li, L. Zhu, Y. Niu, Y. Zhou, Y. Liu, Young exosome bio-nanoparticles restore aging-impaired tendon stem/progenitor cell function and reparative capacity. *Adv. Mater.* **35**, e2211602 (2023).

60. Q. Lei, F. Gao, T. Liu, W. Ren, L. Chen, Y. Cao, W. Chen, S. Guo, Q. Zhang, W. Chen, H. Wang, Z. Chen, Q. Li, Y. Hu, A. Y. Guo, Extracellular vesicles deposit *PCNA* to rejuvenate aged bone marrow-derived mesenchymal stem cells and slow age-related degeneration. *Sci. Transl. Med.* **13**, eaaz8697 (2021).

61. J. Deng, X. Wang, W. Zhang, L. Sun, X. Han, X. Tong, L. Yu, J. Ding, L. Yu, Y. Liu, Versatile hypoxic extracellular vesicles laden in an injectable and bioactive hydrogel for accelerated bone regeneration. *Adv. Funct. Mater.* **33**, 2211664 (2023).

62. A. Liu, D. Lin, H. Zhao, L. Chen, B. Cai, K. Lin, S. G. F. Shen, Optimized BMSC-derived osteoinductive exosomes immobilized in hierarchical scaffold via lyophilization for bone repair through Bmpr2/Acvr2b competitive receptor-activated Smad pathway. *Biomaterials* **272**, 120718 (2021).

63. M. Arra, G. Swarnkar, K. Ke, J. E. Otero, J. Ying, X. Duan, T. Maruyama, M. F. Rai, R. J. O'Keefe, G. Mbalaviele, J. Shen, Y. Abu-Amer, LDHA-mediated ROS generation in chondrocytes is a potential therapeutic target for osteoarthritis. *Nat. Commun.* **11**, 3427 (2020).

64. X. Hong, J. Isern, S. Campanario, E. Perdiguero, I. Ramírez-Pardo, J. Segalés, P. Hernansanz-Agustín, A. Curtabbi, O. Deryagin, A. Pollán, J. A. González-Reyes, J. M. Villalba, M. Sandri, A. L. Serrano, J. A. Enríquez, P. Muñoz-Cánoves, Mitochondrial dynamics maintain muscle stem cell regenerative competence throughout adult life by regulating metabolism and mitophagy. *Cell Stem Cell* **29**, 1298–1314.e10 (2022).

65. G. C. Kujoth, A. Hiona, T. D. Pugh, S. Someya, K. Panzer, S. E. Wohlgemuth, T. Hofer, A. Y. Seo, R. Sullivan, W. A. Jobling, J. D. Morrow, H. Van Remmen, J. M. Sedivy, T. Yamasoba, M. Tanokura, R. Weindruch, C. Leeuwenburgh, T. A. Prolla, Mitochondrial DNA mutations, oxidative stress, and apoptosis in mammalian aging. *Science* **309**, 481–484 (2005).

66. S. E. Schriner, N. J. Linford, G. M. Martin, P. Treuting, C. E. Ogburn, M. Emond, P. E. Coskun, W. Ladiges, N. Wolf, H. Van Remmen, D. C. Wallace, P. S. Rabinovitch, Extension of murine life span by overexpression of catalase targeted to mitochondria. *Science* **308**, 1909–1911 (2005).

67. P. Tian, L. Zhao, J. Kim, X. Li, C. Liu, X. Cui, T. Liang, Y. Du, X. Chen, H. Pan, Dual stimulus responsive borosilicate glass (BSG) scaffolds promote diabetic alveolar bone defectsrepair by modulating macrophage phenotype. *Bioact. Mater.* **26**, 231–248 (2023).

68. Q. Zhai, X. Chen, D. Fei, X. Guo, X. He, W. Zhao, S. Shi, J. J. Gooding, F. Jin, Y. Jin, B. Li, Nanorepairers rescue inflammation-induced mitochondrial dysfunction in mesenchymal stem cells. *Adv. Sci.* **9**, 2103839 (2022).

69. D. Ryu, L. Mouchiroud, P. A. Andreux, E. Katsyuba, N. Moullan, A. A. Nicolet-dit-Félix, E. G. Williams, P. Jha, G. Lo Sasso, D. Huzard, P. Aebischer, C. Sandi, C. Rinsch, J. Auwerx, Urolithin A induces mitophagy and prolongs lifespan in C. elegans and increases muscle function in rodents. *Nat. Med.* **22**, 879–888 (2016).

70. W. Liu, L. Li, Y. Rong, D. Qian, J. Chen, Z. Zhou, Y. Luo, D. Jiang, L. Cheng, S. Zhao, F. Kong, J. Wang, Z. Zhou, T. Xu, F. Gong, Y. Huang, C. Gu, X. Zhao, J. Bai, F. Wang, W. Zhao, L. Zhang, X. Li, G. Yin, J. Fan, W. Cai, Hypoxic mesenchymal stem cell-derived exosomes promote bone fracture healing by the transfer of miR-126. *Acta Biomater.* **103**, 196–212 (2020).

71. M. P. Baar, R. M. C. Brandt, D. A. Putavet, J. D. D. Klein, K. W. J. Derks, B. R. M. Bourgeois, S. Stryeck, Y. Rijksen, H. van Willigenburg, D. A. Feijtel, I. van der Pluijm, J. Essers, W. A. van Cappellen, W. F. van Ijcken, A. B. Houtsmuller, J. Pothof, R. W. F. de Bruin, T. Madl, J. H. J. Hoeijmakers, J. Campisi, P. L. J. de Keizer, Targeted apoptosis of senescent cells restores tissue homeostasis in response to chemotoxicity and aging. *Cell* **169**, 132–147.e16 (2017).

72. M. Ogrodnik, S. Miwa, T. Tchkonia, D. Tiniakos, C. L. Wilson, A. Lahat, C. P. Day, A. Burt, A. Palmer, Q. M. Anstee, S. N. Grellscheid, J. H. J. Hoeijmakers, S. Barnhoorn, D. A. Mann, T. G. Bird, W. P. Vermeij, J. L. Kirkland, J. F. Passos, T. von Zglinicki, D. Jurk, Cellular senescence drives age-dependent hepatic steatosis. *Nat. Commun.* **8**, 15691 (2017).

73. R.-M. Laberge, Y. Sun, A. V. Orjalo, C. K. Patil, A. Freund, L. Zhou, Samuel C. Curran, A. R. Davalos, K. A. Wilson-Edell, S. Liu, C. Limbad, M. Demaria, P. Li, G. B. Hubbard, Y. Ikeno, M. Javors, P.-Y. Desprez, C. C. Benz, P. Kapahi, P. S. Nelson, J. Campisi, MTOR regulates the pro-

tumorigenic senescence-associated secretory phenotype by promoting IL1A translation. *Nat. Cell Biol.* **17**, 1049–1061 (2015).

74. F. Rodier, J.-P. Coppé, C. K. Patil, W. A. M. Hoeijmakers, D. P. Muñoz, S. R. Raza, A. Freund, E. Campeau, A. R. Davalos, J. Campisi, Persistent DNA damage signalling triggers senescence-associated inflammatory cytokine secretion. *Nat. Cell Biol.* **11**, 973–979 (2009).

75. Y. Rinkevich, G. G. Walmsley, M. S. Hu, Z. N. Maan, A. M. Newman, M. Drukker, M. Januszyk, G. W. Krampitz, G. C. Gurtner, H. P. Lorenz, I. L. Weissman, M. T. Longaker, Identification and isolation of a dermal lineage with intrinsic fibrogenic potential. *Science* **348** (2015).

76. J. Reimand, R. Isserlin, V. Voisin, M. Kucera, C. Tannus-Lopes, A. Rostamianfar, L. Wadi, M. Meyer, J. Wong, C. Xu, D. Merico, G. D. Bader, Pathway enrichment analysis and visualization of omics data using g:Profiler, GSEA, Cytoscape and EnrichmentMap, *Nat. Protoc.* **14**, 482–517 (2019).

77. A. Liberzon, C. Birger, H. Thorvaldsdóttir, M. Ghandi, Jill P. Mesirov, P. Tamayo, The Molecular Signatures Database Hallmark Gene Set Collection. *Cell Syst.* **1**, 417–425 (2015).

78. G. Xie, L. Wang, T. Chen, K. Zhou, Z. Zhang, J. Li, B. Sun, Y. Guo, X. Wang, Y. Wang, H. Zhang, P. Liu, J. K. Nicholson, W. Ge, W. Jia, A metabolite array technology for precision medicine. *Anal. Chem.* **93**, 5709–5717 (2021).
